# Connecting the dots in the zona incerta: A study of neural assemblies and motifs of inter-area coordination in mice

**DOI:** 10.1016/j.isci.2023.108761

**Published:** 2023-12-16

**Authors:** Fabrizio Londei, Giulia Arena, Lorenzo Ferrucci, Eleonora Russo, Francesco Ceccarelli, Aldo Genovesio

**Affiliations:** 1 Department of Physiology and Pharmacology, Sapienza University of Rome, Piazzale Aldo Moro 5, 00185 Rome, Italy; 2 PhD Program in Behavioral Neuroscience, Sapienza University of Rome, Rome, Italy; 3 The BioRobotics Institute, Department of Excellence in Robotics and AI, Scuola Superiore Sant’Anna, 56127 Pisa, Italy

**Keywords:** Biological sciences, Neuroscience, Techniques in neuroscience

## Abstract

The zona incerta (ZI), a subthalamic area connected to numerous brain regions, has raised clinical interest because its stimulation alleviates the motor symptoms of Parkinson’s disease. To explore its coordinative nature, we studied the assembly formation in a dataset of neural recordings in mice and quantified the degree of functional coordination of ZI with other 24 brain areas. We found that the ZI is a highly integrative area. The analysis in terms of “loop-like” motifs, directional assemblies composed of three neurons spanning two areas, has revealed reciprocal functional interactions with reentrant signals that, in most cases, start and end with the activation of ZI units. In support of its proposed integrative role, we found that almost one-third of the ZI’s neurons formed assemblies with more than half of the other recorded areas and that loop-like assemblies may stand out as hyper-integrative motifs compared to other types of activation patterns.

## Highlights

• Assembly analysis reveals that the ZI coordinates with multiple regions
• Loop-like assemblies embody a specific motif of inter-area coordination
• ZI cells forming loop-like assemblies act as hub neurons
• ZI forms asymmetric loop-like motifs of coordination mostly reentering the ZI

## Graphical abstract

**Figure:**
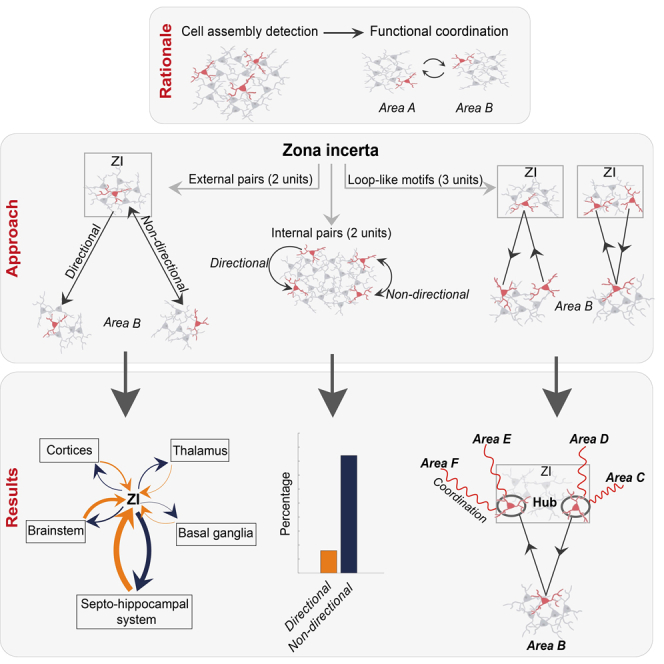

## Introduction

The most enigmatic and elusive of the subthalamic structures is the zona incerta (ZI), first identified by Forel as the area of the brain “of which nothing certain can be said.”^1^ Despite numerous efforts to shed light on the behavioral and cognitive aspects of this area’s processing, the overall picture is still hazy. However, it is known that it establishes a wide range of mostly GABAergic and marginally glutamatergic anatomical connections with the entire neuraxis,^1^^,^^2^ suggesting that its outputs may exert different effects on the targeted regions based on the neurotransmitters and neuropeptides released by its projections. Although its function is far from clear, it is thought that the ZI serves as an integration center for the multiple interoceptive and exteroceptive multisensory stimuli it receives from various centers to promote and modulate a wide range of adaptive behaviors.^1^^,^^3^ Additionally, the promising results obtained by the deep brain stimulation (DBS) on the ZI for treating motor symptoms associated with Parkinson’s disease^4^ have raised interest in this subthalamic structure and bolstered the research on it.

To shed light on the integrative role of the ZI, in this study we investigate its interaction patterns with a large network of simultaneously recorded cortical and subcortical regions. Functional interaction can be inferred from data collected with different methods, such as magnetic resonance imaging (MRI) data^5^ or neurophysiological data^6^^,^^7^^,^^8^^,^^9^ which can be analyzed for coordinated activity through many computational tools today available.^10^^,^^11^^,^^12^ Here we focus on the identification of cross-regional cell assemblies, groups of neurons recorded in different brain regions which share a coordinated pattern of activation and might thus be the signature of inter-regional interaction.^13^^,^^14^

First introduced by Donald Hebb in 1949,^15^ cell assemblies form a functional unit of computation that might reflect internal processes, encode memories, or respond to perceptual experiences. The transient and flexible coordination of the single units taking part in assemblies expands the coding capacity of a network. Single units can, in fact, take part in multiple assemblies and thus encode different information at different moments in time. Moreover, the extraction of information from the coordinated activity of a network also opens perspectives in the field of brain-machine interfaces.^16^

Together with the computational advancements which make now possible the detection of cell assemblies with multiple methods,^17^ recording techniques have also made substantial progress in the recent years: while a decade ago recording more than a dozen neurons simultaneously was an unsustainable challenge and relied mostly on the study of the cross-correlations between a small number of pairs of neurons,^18^^,^^19^^,^^20^^,^^21^^,^^22^^,^^23^ today, especially thanks to cutting-edge tools such as the Neuropixels probes,^24^^,^^25^ we have access to large datasets of brain-wide recordings covering hundreds or even thousands of neurons simultaneously. This offers a unique opportunity to investigate inter-area coordination at an unprecedented range, enabling us to answer questions on a larger scale.^9^

In this manuscript we will quantify the degree of coordination between brain areas by detecting cross-regional assemblies. In particular, the lag between the activation of assembly units recorded in different brain regions will inform us about possible directions of information flow. Additionally, by identifying assemblies of more than two neurons we can search for network motifs designed as a reoccurring pattern of interaction. Motifs, stereotypical patterns of connectivity, have been described and computationally studied in multiple fields,^26^^,^^27^ including neuroscience.^28^^,^^29^^,^^30^ However, they have been extensively studied mainly in simple organisms, such as the *Drosophila*.^31^ As pointed out by Alon,^32^ “Evolution seems to have converged on the same motifs in different systems and different organisms, suggesting that they are selected for again and again on the basis of their biological functions.” Recent studies have employed types of loop-like motifs as key for investigating brain connectivity. This approach, using techniques like functional MRI (fMRI)^33^ and electroencephalogram (EEG),^34^ has been found to hold potential for gaining a more comprehensive understanding of the connections between different brain regions. Here, we address the study of motifs in the ZI at the single cell level by examining loop-like assemblies, specific motifs composed of sequential activation of three neurons, in which the first and last units of the chain belong to a same brain region while the middle unit belongs to a different region. Loop-like assemblies could underpin loops of functional recurrent coordination between brain areas that might suggest the presence of reentrant, or feedback, signals in the ZI. In addition to identifying loop-like assemblies, we also aim to gain a deeper understanding of the integrative nature of the ZI by examining the inter-area coordination of single ZI neurons. Specifically, our goal is to determine whether ZI functional coordination is widespread, covering a wide range of brain regions, or more selective, targeting a smaller number of areas.

## Results

### Analysis of neural pairs

A total of 289 cells were recorded in the ZI across 2 mice and 4 sessions (as shown in Table S1), together with recordings of 24 external brain areas (Figure S1). We selected and used for all analysis only neurons with more than 100 spikes, resulting in the removal of 2 neurons from the caudoputamen (CP).

**Figure 1:**
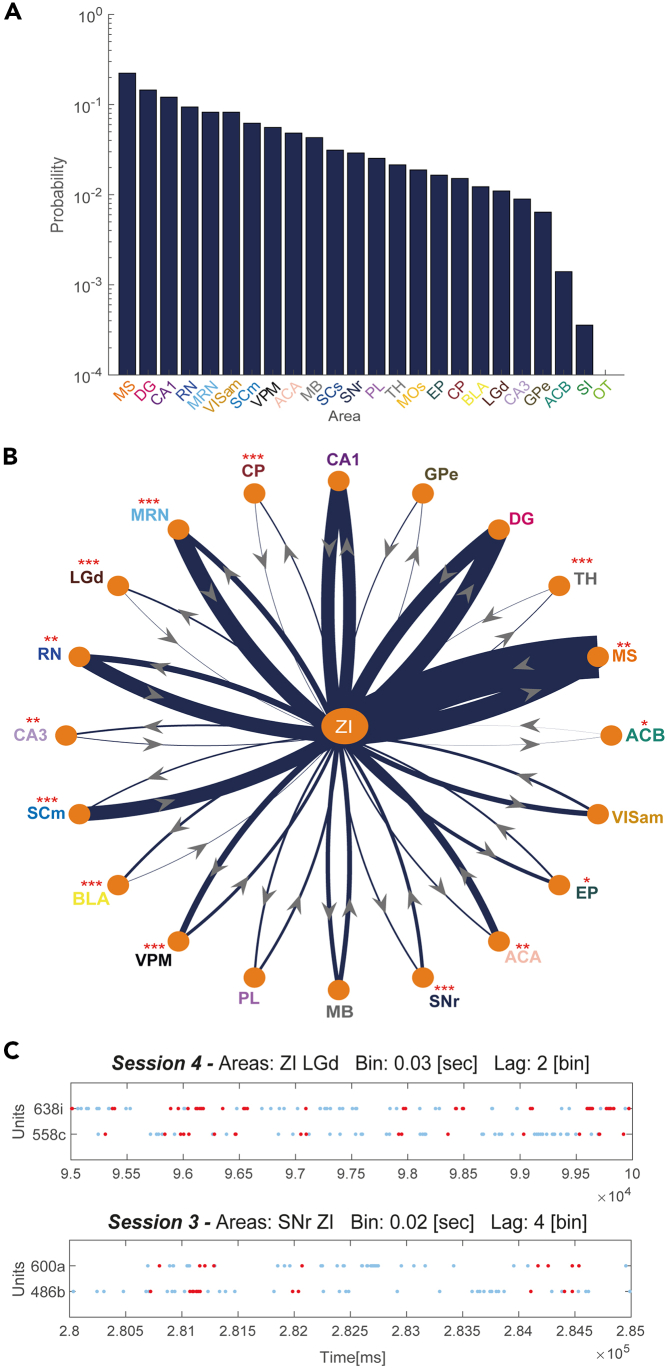
Pair assemblies analysis reveals asymmetrical coordinative relationships (A) Histogram on log scale showing the ranking of the areas according to their probability of forming pair assemblies with ZI, regardless of the type of assembly (directional and non-directional). (B) Directed graph representing the functional relationship established by the ZI with the areas simultaneously recorded by Steinmetz and colleagues.^35^ Each node represents an area, while the thickness of the directed edges reflects the probability of finding assemblies in the two possible directions (to and from the ZI). ∗ indicates a significant asymmetry in the occurrence of assemblies to and from ZI (binomial test with hypothesized probability of 0.5). ∗p < 0.05; ∗∗p < 0.01 ∗∗∗p < 0.001. For the exact p-values, please refer to Table S2. (C) Raster plots of two example external assemblies. Each dot indicates an action potential, light blue dots indicate all the spikes of the two neurons composing the assembly, and red dots mark the spikes fired by the units during the assembly activation. The upper panel shows an assembly detected at a 30 ms temporal resolution with a 60 ms lag between the activation of the ZI unit and the following LGd unit. The lower panel shows the activity of an assembly detected with a 20 ms temporal resolution and a lag of 80 ms between the activation of the SNr unit and the following ZI unit.

Figure 1A shows the ranking of the areas according to their probability of forming external assemblies with the ZI, regardless of whether they are directional or not. Figure 1B, instead, shows the probability of occurrence of directional assemblies between the ZI and other regions, divided by directionality. In the figure, the thickness of the edges linking the ZI with another area is proportional to the probability of detecting “from ZI” assemblies (centrifugal directionality of the edges) and “to ZI” assemblies (centripetal directionality). Thicker lines denote strong functional coupling in a specific direction that could be interpreted as a strong functional and directional flow of information from/to the ZI.

We compared the number of assemblies “from ZI” with those “to ZI” to identify an eventual preference in the directionality of interaction. To assess the statistical significance of the difference in the occurrence of the two assembly directionalities, we applied a binomial test (see STAR methods).

We found several regions forming assemblies with ZI with a preferred directionality (Figures 1B; Table S2; Figure S2 shows for the same analyses the fraction of synchronous assemblies). As shown by Table S2, the preference in directionality changed according to the target region. For instance, the “from ZI” category prevailed with a statistically significant difference (p < 0.05) in the couples ZI/nucleus accumbens (ACB), ZI/basolateral amygdala (BLA), ZI/ Ammon’s Horn 3 (CA3), ZI/CP, ZI/endopiriform nucleus (EP), ZI/lateral geniculate nucleus (LGd), ZI/thalamus (TH), and ZI/ventral posteromedial nucleus of the thalamus (VPM), whereas the “to ZI” category prevailed with a statistically significant difference in the couples anterior cingulate area (ACA), midbrain reticular nucleus (MRN), medial septum (MS), red nucleus (RN), superior colliculus motor-related (SCm), substantia nigra pars reticulata (SNr), and ZI. Finally, there was no statistically significant asymmetry in directionality in assemblies involving the ZI and Ammon’s Horn 1 (CA1), dentate gyrus (DG), globus pallidum external segment (GPe), midbrain (MB), prelimbic area (PL), or anteromedial visual cortex (VISam).

Interestingly, some areas appear to rank similarly in the overall propensity to form assemblies with ZI but differentiate in the directionality of such assemblies. For instance, in Figure 1A, MRN and VISam are ranked similarly, but in Figure 1B the edge thickness between MRN and ZI is greater than that between VISam and ZI. This difference results from only the non-directional assemblies being included in the ranking plot. While the probability of detecting directional assemblies is higher for the MRN/ZI pair, in the overall probability of detecting an assembly, the paucity of directional assemblies between ZI and VISam is balanced by a large probability of finding synchronous assemblies (see Table S3 for further details).

Additionally, Figure 1B also shows that the incertal functional coordination with the basal ganglia (BGs) is somewhat scarce, except for SNr which was only slightly more functionally related to the ZI than GPe, CP, and ACB. On the contrary, a strong functional coordination of the ZI with the MS and hippocampus was evident, since several hippocampal fields (the DG and CA1) and the MS occupy the first positions in the ranking (Figure 1A). However, a statistically significant preference in directionality was only detectable in the relationship between the ZI and MS but not in ZI/CA1 and ZI/DG (Table S2).

To assess the reliability of the obtained results, a circular shuffling procedure was repeated 10 times for each possible couple of areas containing the ZI in each recording session (see STAR methods). The results obtained on each couple were then normalized with respect to the number of possible pairs of elements on that set of neurons. On average, in the shuffled sets we obtain a probability to find assemblies of 1.5 × 10^−5^, with a maximum value of 1.3 × 10^−4^. Note that we never identified more than 2 assemblies in a single run of the algorithm on the shuffled sets. As we expected, since the algorithm has been extensively tested,^36^ this finding confirms that it is highly unlikely that the assemblies identified by *CADopti* are the result of chance-related correlations.

To visually appreciate the coordination in activity of the cells composing the assemblies, we plotted the rasters of the activity of two examples of assemblies, the first constituted by a leading incertal neuron followed by the activation of a trailing neuron located in the LGd and the second constituted by the leading neuron belonging to the SNr and the trailing neuron to the ZI (Figure 1C). The upper panel refers to an assembly characterized by a temporal structure in which the delay (*Bin* x *Lag*) between the activation of the two units was 2 × 0.03 s = 60 ms, while the lower panel refers to an assembly with a delay of 4 × 0.02 s = 80 ms.

To further characterize the interaction between the ZI and the other areas, we then plotted the distribution of the delay between the reciprocal activations of the neurons of external pair assemblies (Figures S3–S5). Negative values indicate “to ZI” assemblies, positive values indicate “from ZI” assemblies, and values equal to zero indicate synchronous assemblies. In the ZI/LGd, ZI/ACA, and ZI/secondary motor area (MOs) pairs, we observed a very specific profile of functional coordination, with a high probability of forming non-directional assemblies and, at the same time, a high probability of finding a specific type of directional assembly, whether from or to the ZI depending on the combination of areas. In the case of ZI/VPM, ZI/CP, ZI/TH, and ZI/VISam, we could identify almost only synchronous assemblies, while the ZI/MS pair displays a unique profile of temporal structures of functional coordination, with a wide distribution around the 0 lag that remains itself the most represented case. For ZI/GPe and ZI/MRN, the directional assemblies were the most represented, while the ZI/EP pair stands as the only example of areas with almost only directional assemblies.

We also computed what we designed as the *Int-Ext index*, which measures how likely the ZI is to form assemblies internally compared to how likely it is to form assemblies externally (for more information, please see STAR methods).

Figure S6 shows that ZI neurons have a higher probability of forming external than internal assemblies with only the hippocampus and the MS.

**Figure 2:**
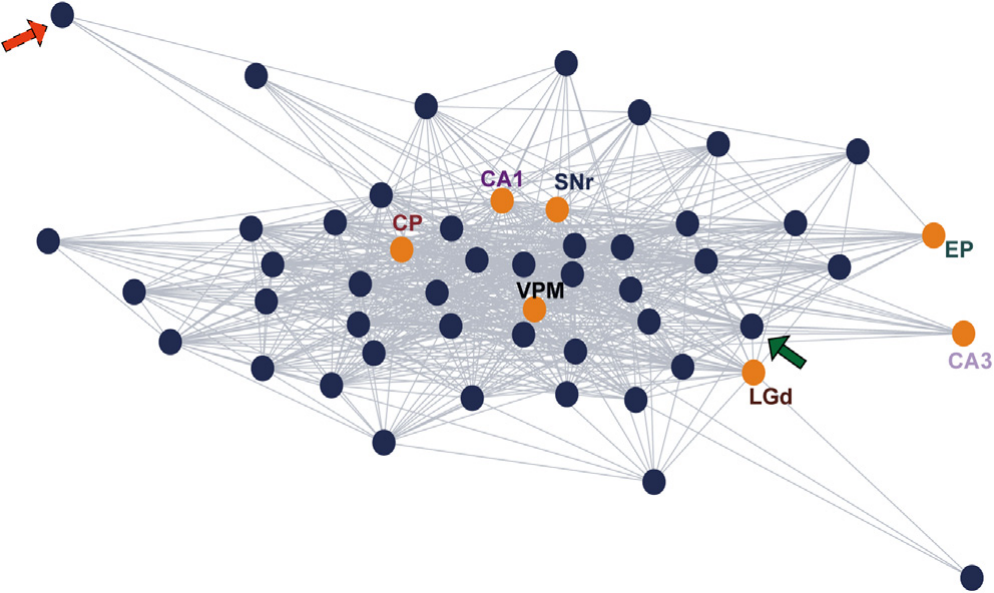
Assembly analysis on single cells shows multiple degrees of integrative ability Undirected graph representing functional interactions both between incertal neurons (blue dots) and between each incertal neuron and the entire external areas (orange dots) for one example session (Session 4). The red arrow with dotted borders indicates an incertal neuron forming assemblies (edges) with one external area only, while the green arrow with continuous borders shows an incertal neuron establishing assemblies with all external areas. Both indicated neurons also form assemblies with other incertal cells.

Since *CADopti* works at a single-neuron level of resolution, we decided to pixelate the entire ZI into its constituent neurons in order to investigate the coordination of individual incertal cells with other incertal cells and with entire external areas. In Figure 2, we present an example session in which blue dots represent incertal neurons and orange dots represent external areas recorded in that particular session. Interestingly, some neurons were characterized by a very high degree of coordination with multiple areas: for example, the cell indicated by the green arrow (continuous borders) formed assemblies with all external regions and with internal incertal neurons. On the contrary, some cells formed assemblies only with a few areas, or even in some cases with no external areas, by functionally interacting only internally with other neurons of the ZI. For example, the cell indicated by the red arrow (dotted borders) formed assemblies with CP only.

**Figure 3:**
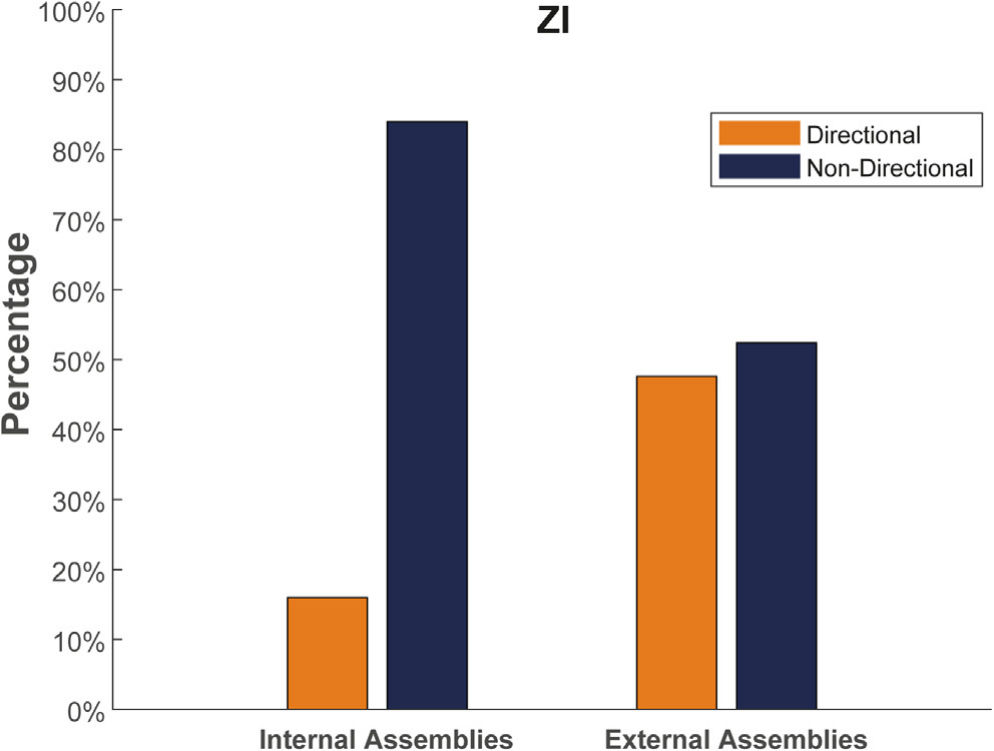
Asymmetrical distribution of synchronous and asynchronous patterns between internal and external assemblies Histogram showing the percentage of detected directional and non-directional assemblies either internal to the ZI or external between the ZI and other areas. The results pool assemblies across all sessions, as described in the STAR methods section.

Given our interest in the intrinsic patterns of coordination within ZI, we detected assemblies between incertal neurons, limiting our search to assemblies constituted by two neurons, which we distinguished into “directional” and “non-directional” (synchronous) assemblies. Figure 3 shows the percentage of directional and non-directional assemblies in pairs identified within the ZI and between the ZI and other regions. While in both cases the number of detected non-directional assemblies surpasses that of the directional ones, this discrepancy was substantially higher in within-region assemblies. While intuitive, the tendency to an enhanced synchrony when selecting within-region assemblies is not the only possible outcome of this analysis. In fact, in the MS, we found a prevalence of directional assemblies among the ones detected within the region (Figure S7).

**Figure 4:**
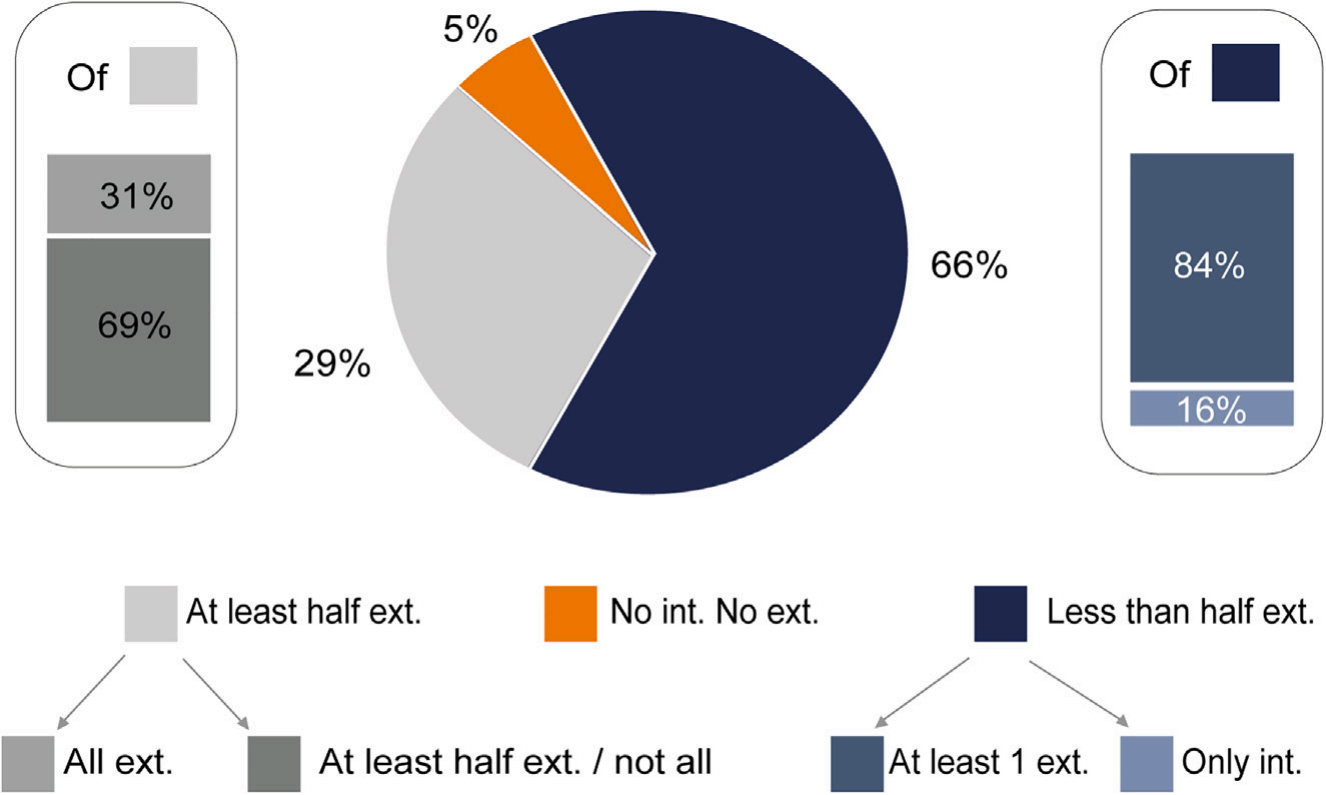
Percentage of cells forming assemblies with different proportion of areas Percentage of incertal neurons forming no assembly (orange) or forming at least one assembly with at least half (gray) or less than half (blue) of the other simultaneously recorded regions. The terms "ext" and "int" refer to the formation of assemblies with external and internal areas, respectively. Incertal cells forming pairs with less than half of the external areas recorded in the same session are referred to as "less than half ext." In turn, these divide between cells forming assemblies only with other neurons of ZI ("Only int.") and cells forming assemblies with at least an external region ("At least 1 ext."). Incertal cells forming pairs with more than half of the external areas are referred to as "At least half ext." These divide into "All ext.", forming assemblies with at least one unit in each of all other regions, and "At least half ext./not all", forming assemblies with at least half but not all the areas simultaneously recorded in the same session. Cells that do not form assemblies either internally or externally are labeled as "No int. No ext.".

To evaluate the degree of global coordination of ZI with the rest of the brain, we next computed the proportion of incertal neurons forming assemblies with the multiple recorded areas. As shown by Figure 4, approximately 29% of the incertal neurons formed assemblies with at least half of the external regions recorded in each session, a number which varied, according to the session, between 6 and 13 regions. Interestingly, approximately one-third of these neurons formed assemblies with all the external regions simultaneously recorded. On the other hand, 66% of the incertal neurons formed assemblies with less than half of the external regions. Of these, approximately 16% did not form assemblies external to the ZI. Lastly, about 5% of the incertal neurons formed neither internal nor external assemblies, although this percentage is probably overestimated because these cells might still form assemblies with unrecorded incertal or external cells.

We need to point out that these proportions should be taken with caution because they depend on the areas studied and cannot be generalized to other areas. It is certainly plausible that a neuron with high specificity when considered for its coordinative relationships with the areas considered in this study might then be functionally coordinated more broadly with other areas not studied in this work.

### Analysis of loop-like triplets

**Figure 5:**
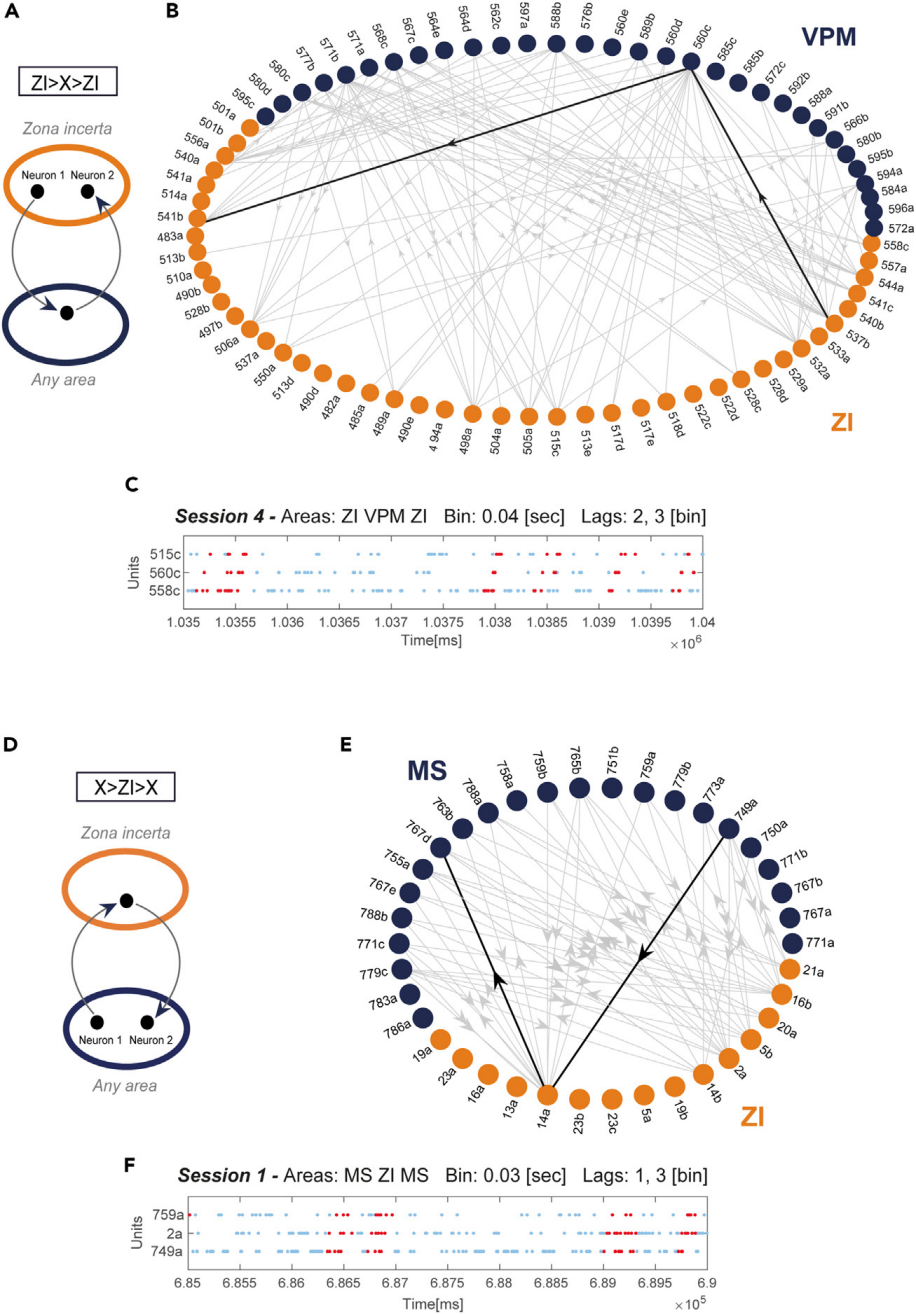
Examples of loop-like triplets possessing different structures (A) Scheme showing one of the two possible structures of loop-like triplets, where the first and last active neurons belong to the ZI and the second one belongs to another area X of those tested (ZI→X→ZI). The first and last units are always different neurons. (B) Directed graph with all loop-like assembly triplets detected between incertal (orange dots) and VPM (blue dots) neurons recorded simultaneously in session 4. The highlighted edges show an example of loop-like triplet with the same structure exemplified in (A). (C) Raster plot showing an example of loop-like triplet spanning the ZI and the VPM, with the aforementioned structure; light blue dots correspond to all spikes fired by the assembly units, and red dots correspond to spikes fired during the assembly activation. (D) Scheme of the alternative loop-like structure, X→ZI→X. (E) Directed graph with all loop-like triplets detected between incertal (orange dots) and MS (blue dots) neurons recorded simultaneously in session 1. The highlighted edges show an example of loop-like triplet with the same structure exemplified in (D). (F) Raster plot of a loop-like assembly with an MS→ZI→MS structure. To be noted, rasters do not display the assemblies highlighted for the graph representation.

Given the broadness of the functional coordination of the ZI with the other recorded regions (Figure 2), we decided to characterize it further and search for more complex patterns of coordination. Thus, we extended the search to triplets, assemblies composed of three neurons, distributed between ZI and other areas. In particular, we restricted the analysis to what we defined “loop-like” triplets, that is, triplets spanning two areas (A and B) with an A→B→A structure. It follows that two loop-like structures involving ZI are possible: ZI→X→ZI, where the first (leading) and last active neurons of the assembly belong to the ZI and the middle unit belongs to another area, and X→ZI→X with only the intermediate neuron belonging to the ZI. In both cases, the first and last neurons are different units of the same area, as shown in Figures 5A and 5D.

As example of both types of triplets, in Figures 5B and 5E we reported all loop-like triplets detected in a session between ZI and VPM and the ZI and MS, respectively. Units taking part in loop-like triplets are connected by directed edges, which specify the direction of the temporal lag between the activation of the assembly units. In the graphs, marked in black are two examples of ZI→X→ZI triplet (Figure 5B) and X→ZI→X triplet (Figure 5E). The loop-like structures formed by the ZI neurons reveal an organization with a few cells acting as hubs, with a higher number of coordinative relationships compared to the other neurons. For example, in Figure 5E several assemblies are formed with the same neuron 14a and none with the neuron 13a. On the contrary, the loop-like structures in the MS and VPM appear to involve most of the neurons evenly.

In Figures 5C and 5F we show the raster plots of two examples of loop-like triplets, with the leading neuron respectively in the ZI or in the external area.

**Figure 6:**
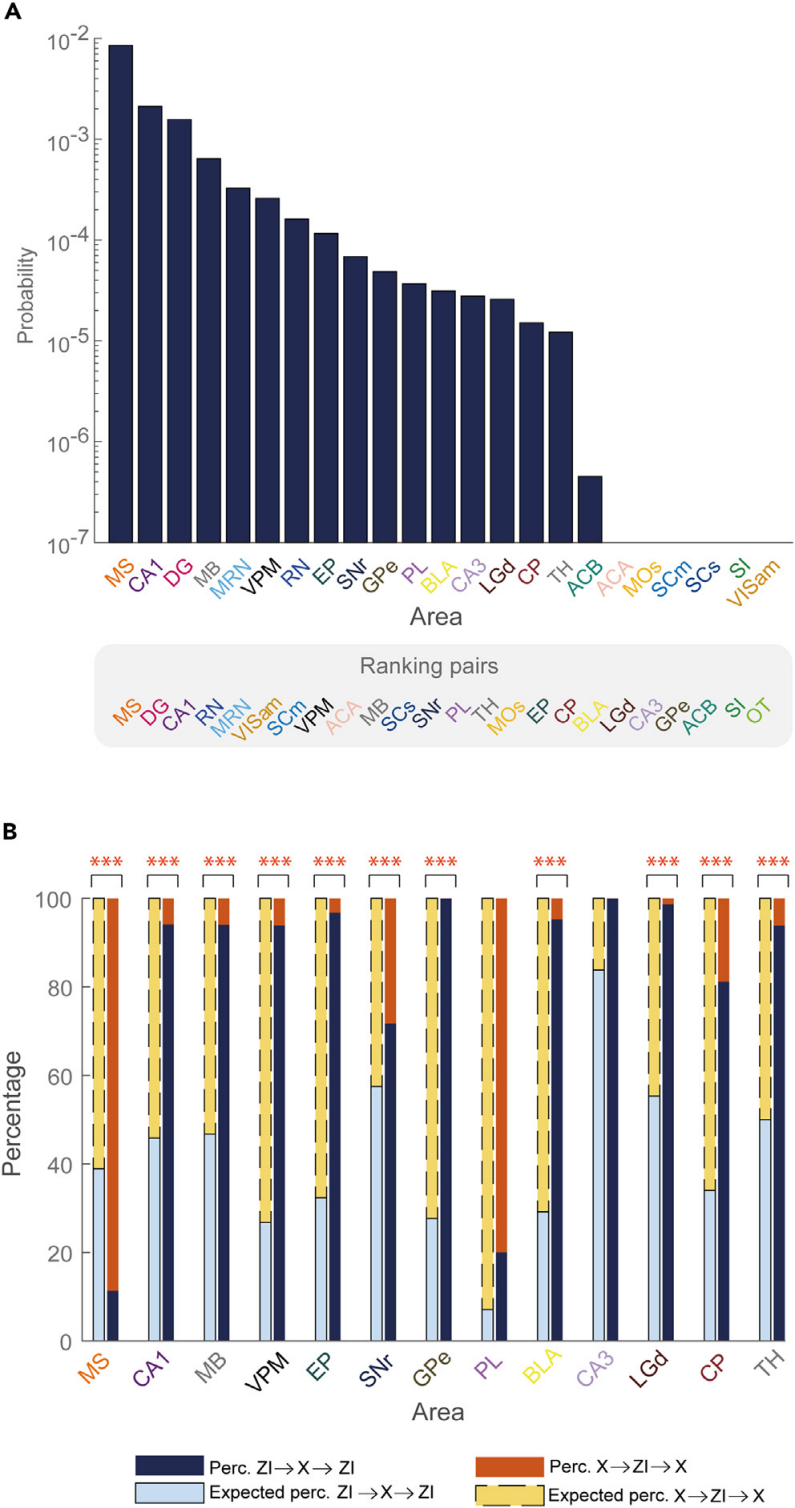
Assembly analysis on loop-like triplets reveals functional signals mostly reentering the ZI (A) Histogram showing the ranking of areas according to the probability of forming loop-like triplets with the ZI, regardless of the type. OT is not included since it does not form pairs with the ZI and thus no triplets can be formed. The lower gray box reports the ranking of areas according to their probability of forming pair assemblies with ZI as reported in Figure 1A for comparison with the upper ranking. (B) Histogram comparing the observed percentage of loop-like triplets of ZI→X→ZI (right bar, dark blue) and X→ZI→X (right bar, orange) types with that expected by chance (left bar, light blue with continuous borders and yellow with dotted borders). Areas forming with the ZI less than 10 loop-like assemblies in total are not included. ∗ indicates loop-like triplets with a significant difference in the probability of forming the two loop-like triplets (binomial test with different hypothesized probabilities depending on the particular area considered). ∗p < 0.05; ∗∗p < 0.01 ∗∗∗p < 0.001. For the exact p-values, please refer to Table S4.

The ranking plot in Figure 6A shows the probability of forming a loop-like triplet, regardless of its structure, between the ZI and the other areas (except for the olfactory tubercle (OT), for which no pairs were detected with the ZI either). The probability of ZI to form loop-like triplets was higher with the MS than with every other area, consistent with the probability of forming pair assemblies (Figure 1A). However, a high probability of forming pairs was not always predictive of a high probability of forming loop-like triplets. For instance, ZI/VISam, ZI/SCm, ZI/superior colliculus sensory-related (SCs), ZI/MOs, and ZI/ACA shared a relatively high probability of forming pairs but not loop-like triplets. On the contrary, while the ZI/EP probability of forming pairs only ranked 16th in Figure 1A, the probability of forming loop-like triplets ranked 8th. A similar discrepancy is observed between the ZI and the GPe, which ranked 21st for the pairs and 10th for the loop-like triplets.

We then evaluated if one of the two types of loop-like structure was more frequent than the other and more frequent than what expected by chance given the number of recorded units in the different regions. The histogram in Figure 6B shows that the detected types of loop-like structures strongly deviate from what we would expect by chance. For example, in CA1 and the midbrain, as in most of the cases, we observed a significantly higher probability of finding ZI→X→ZI than X→ZI→X loop-like triplets. The ZI/MS couple represents an exception, displaying the opposite pattern, with a significantly higher probability of forming the alternative type of loop-like triplets, indicating a prevalence of reentrant activity in the MS than in the ZI. For further details, see Tables S4 and S5.

**Figure 7:**
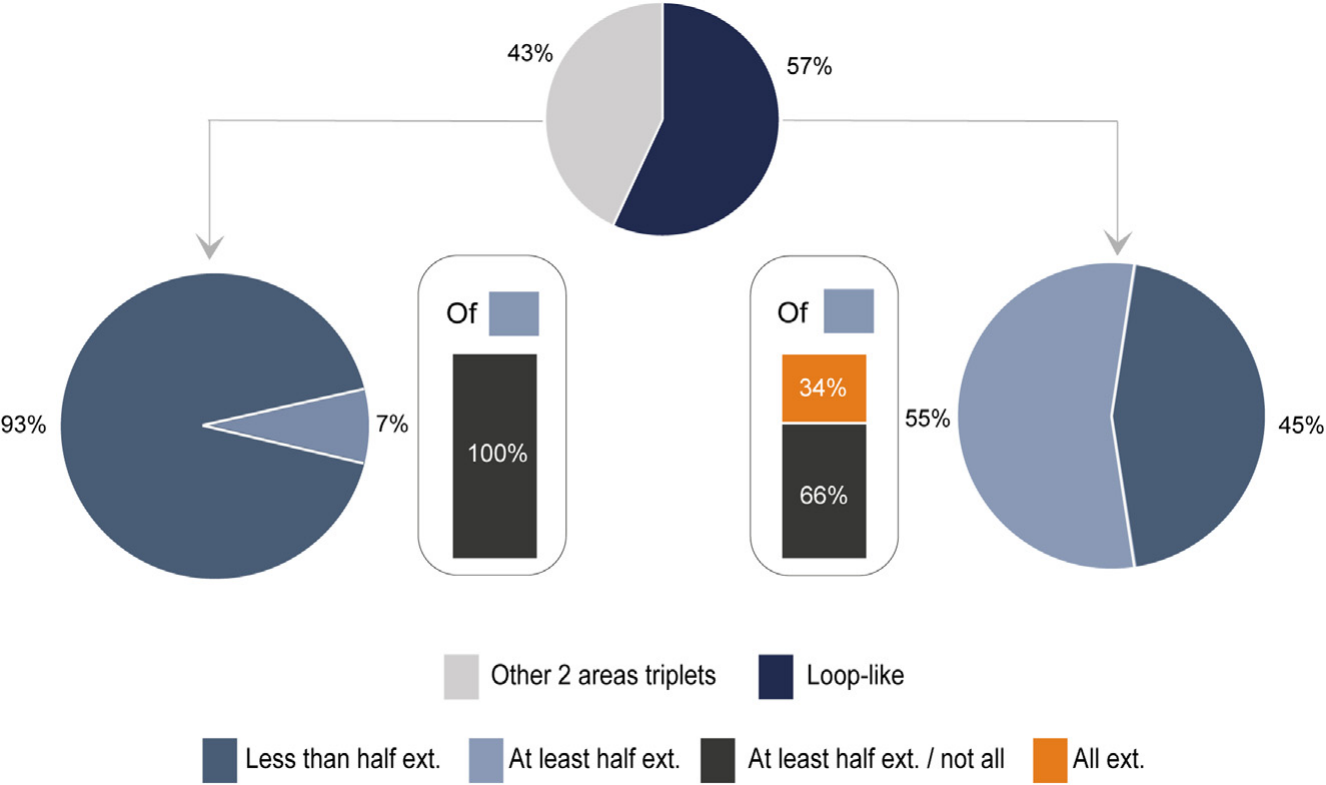
Only neurons forming loop-like triplets form assemblies with all the areas recorded simultaneously The upper pie chart illustrates the percentage of cells that took part in at least one loop-like triplet (shown in blue) and those that took part in non-loop-like triplets only (shown in gray). The lower pie charts represent the percentage of cells of the two categories (non-loop-like triplets on the left, and loop-like triplets on the right) that formed assemblies with various proportions of external areas. These latter proportions are the same as those mentioned in Figure 4.

Furthermore, we decided to extend the analysis of Figure 4 by jointly considering the coordination of single units with other regions together with their membership to loop-like assemblies. We observed that neurons in loop-like triplets constituted nearly the entire population (91%) of neurons forming pairs with at least half of the areas, and only 34% of those forming pairs with less than half of the areas (data not shown). Thus, we wonder whether incertal neurons within loop-like triplets may be more coordinated with external regions even beyond the area involved in the loop. Our results (Figure 7) support this intuition, showing that 55% of neurons in loop-like triplets coordinate to at least half of external areas, while only 7% of neurons in non-loop-like triplets do so. For example, the ZI neuron taking part in the highlighted triplet in Figure 5E were found to have connections with more than half of the 13 recorded areas while neuron 14b was found to have connections to all the areas (not shown).

In an additional analysis, we evaluated whether these neurons are also highly coordinated within the ZI network and not only externally. On average, we found that only 10% of the incertal neurons took part in an assembly with at least half of the other simultaneously recorded ZI neurons. However, when examining ZI neurons in loop-like triplets, this percentage increased to 17%, while it dropped to 4% in neurons belonging only to non-loop-like triplets. This suggests that ZI neurons in loop-like triplets are strongly coordinated both internally and with external areas.

## Discussion

### Pair assemblies

In this study we analyzed the functional coordination of the ZI by quantifying the assemblies formed by its neurons with multiple brain regions involved in motor, sensory, and emotional processing. Our analysis revealed a mix of symmetrical and asymmetrical functional relationships, with specific key neurons being able to coordinate their activity with multiple areas. Notwithstanding the function of such coordination cannot be determined in our study, a possibility is that it reflects information transfer and integration processes. This interpretation is in line with the conspicuous asymmetry of directionality of pairs involving the ZI and the external areas and, interestingly, mirrors the anatomical relationships existing between them. For example, in the light of this interpretation, our results suggest a relationship based on a reciprocal albeit asymmetrical flow of information between the ZI and the SCm, with more “to ZI'' than “from ZI” assemblies (Figure 1B). Previous studies have shown that anatomical interconnections between these regions are a shared mammalian feature.^37^ Specifically, the stimulation of the SC activates the ventral sector of the ZI^38^ and incertal GABAergic neurons have been shown to send dense projections to the SCm^2^ with the overall effect of incertal influence over SCm being predominantly inhibitory.^37^^,^^39^^,^^40^ It is important to note that here, as in the vast majority of the literature on cell assemblies, assemblies were detected by the coordinated activation of simultaneously recorded units. The algorithm is however unable to detect systematic inhibitory relations between neurons. This means that eventual inhibitory projections that would inhibit the activity of target neurons are either missed or captured because of the rebound excitation of the inhibited units. It is possible that this is the case for the ZI/SCm interaction which yields high and low probabilities of detecting, respectively, “to ZI” and “from ZI” assemblies, coherently with the proposed excitatory effect of the SCm on the ZI and the inhibitory effect of the ZI on the SCm. This interpretation is also compatible with the observation that ZI GABAergic neurons receive conspicuous projections from the SCm,^2^ since the activation of collicular cells would produce the activation of target incertal cells, making a possible coordinative relationship between them detectable by the algorithm.

The coordination of the ZI with the MRN and RN exhibits a similar pattern of functional interaction: inhibitory incertal neurons have been shown to target the RN and the MRN,^2^^,^^41^ but the back projections from the RN and MRN to the ZI^42^ have not yet been neurochemically characterized to our knowledge. There is a possibility, albeit speculative, that the high proportion of “to ZI” assemblies may indicate a yet-uncharacterized excitatory effect of these areas over the ZI. Considering the BGs, we found a very loose functional coupling with ZI, specifically in the CP, GPe, and the ACB, except for the SNr, which shows the highest probability of detecting assemblies among the BG, consistent with anatomical findings showing the existence of afferent^43^ and efferent^43^^,^^44^ projections between ZI and SNr, which are also denser than interconnections between the ZI and the GP or CP.^43^ Examining the sensory-related areas, our study shows that the functional coupling between the ZI and the VISam is stronger than the coupling with the LGd and the SCs (Figure 1A). Interestingly, the high rank positioning of the VISam in the probability to form assemblies (Figure 1A) was not associated with a sharp directionality (Figure 1B) but due to a high proportion of synchronous assemblies that could be accounted for, for example, by the simultaneous activation of both areas due to a common input. According to Power et al.,^45^ the ZI receives sparse afferents from the LGd, the primary thalamic relay nucleus for visual information,^46^ which is consistent with the low probability of finding assemblies between the LGd and the ZI. More controversial is the magnitude of the incertal anatomical connections with the VPM, with some studies reporting the absence or scarcity of incertal terminals in this nucleus^38^^,^^47^^,^^48^ and some reporting robust labeling in the VPM following anterograde and retrograde tracer injections in the ZI.^49^^,^^50^ Our result argues in favor of a functional interaction between these areas, showing a modest probability of forming assemblies between the ZI and the VPM.

Regarding limbic areas, a very poor characterization is nowadays available regarding the relationship between the ZI and the hippocampus or the medial septum: it is known that very few neurons result labeled after retrograde trace injections in the hippocampus^51^ and that the medial ZI projects to the MS.^52^ Yet, no backward projections have been identified, and no overall effect of incertal inputs on those areas has been defined to our knowledge. Nonetheless, our results reveal a remarkable coordination between the ZI and hippocampal and septal areas, the meaning of which remains unclear.

We next sought to characterize the ability of the incertal neurons to form directional and non-directional assemblies internally, and, for comparison, we ran the same analysis on the MS, which shows the highest degree of coordination with the ZI. We anticipated that non-directional internal assemblies would be more common in each area since, within a region, we presume a higher redundancy in coding, and thus synchronous firing, than between two regions. However, it was surprising to see that, in contrast to the ZI and to most of the other areas, the MS showed a preference for internal assemblies with lag as opposed to the synchronous ones. This pattern of activity would correspond to the presence of strong sequential activations within MS. This finding highlights that different areas can display different stereotypical patterns of activity reflecting different information-processing mechanisms.

Lastly, we characterized the degree of coordinative ability of each incertal neuron and found that 9% of these cells coordinate their activity with at least one neuron of every other area simultaneously recorded. Albeit the possibility that such units may be phase-locked to an oscillation shared among all the areas cannot be ruled out, in the light of the role of the ZI as an integrative node for the modulation of a rich range of adaptive behaviors,^1^^,^^3^ it is tempting to hypothesize that the coordination of these incertal neurons to neurons belonging to different areas may reflect an integrative process and a cross-regional exchange of information, and, thus, such neurons would represent the ones with the highest integrative ability. However, future studies are warranted to further define the meaning of these brain-wide coordinative relationships involving the ZI.

### Loop-like assemblies

We searched for assembly structures representing a specific three-node motif or connected subgraph of three neurons distributed among couples of areas, as explained by Figure 5A, which we labeled “loop-like triplets”. Motifs can emerge from broader networks of anatomically connected nodes when specific subsets of them become functionally engaged in a certain process.^26^^,^^28^ The loop-like assembly corresponds to graph type number 2 of the list of graphs described by Milo et al.^26^ in their Figure 1B. Motifs have usually been investigated from both a structural and a functional perspective, regardless of the neuroanatomical locations of the neurons composing the network. In this context, the main novelty of our approach is the search for motifs across areas. Surprisingly, for most of the analyzed regions, we found an asymmetrical probability of having the two types of loop-like triplets including the ZI. Notably, the CA1 of the hippocampus and the MS both stood out as the most functionally linked areas to the ZI but displayed different loop-like types of motifs. The ZI/CA1 couple displayed the ZI→X→ZI loop-like motif more often than expected by chance, while the ZI/MS couple favored the X→ZI→X loop-like structure instead. We observed that even couples of areas that, at the assembly pair level, show no significant asymmetry in preferred directionality, at the loop-like level, can display prominent differences in the probability of forming the two alternative structures, as for the ZI/CA1 couple. This finding suggests that the directionality of the pairs and the asymmetries of the loop-likes structures are two independent features. Additionally, loop-like assemblies can be found only between two functionally related areas, capable of forming at least assemblies of two neurons. However, the presence of pair assemblies alone does not necessarily mean that loop-like triplets will be produced. This is because, even if two regions are linked by pair assemblies of both directionalities, triplet assemblies will not emerge unless the pairs hinge on the same set of neurons and activate in a coordinated fashion themselves. For instance, even though the RN and the ZI establish directional pairs of both directionalities with a relatively high probability, the probability of forming loop-like triplets is still very low. On the contrary, the GPe, EP, and the ZI showed a relatively low probability of forming pairs when compared to other couples of areas, but loop-like triplets occurred with a higher probability than in other couples, suggesting that loop-like triplets may embody particular types of information-processing mechanisms outside of the reciprocity of their interaction.

Multiple network mechanisms might be at the origin of loop-like activation structure. As in the case of pairs, one possible source of coordination across brain regions comes from the shared entrainment on a common oscillation.^14^ In the case of loop-like activation structures, the first and last unit of the triplet could be active in two consecutive oscillation cycles, and the intermediate unit phase-locked on the same oscillation but at a different phase.

Alternatively, region coordination could arise from excitatory drives (either direct or through disinhibition), and loop-like triplets could represent a particular type of reentry of information, or recurrent activity, which, even in the absence of monosynaptic anatomical connectivity, has been shown to characterize processes connected with the suppression of alternative plans or conflicting alternative patterns of activity, binding sensory features, or support long term memory.^53^ Moreover, the observation that all the neurons coordinating their activity with all the areas simultaneously recorded enter as members of a loop-like assembly raises the possibility that these specific types of three-neurons assemblies may embody integration-oriented motifs of functional coordination. Further studies are needed to determine if the characteristic higher coordination with many brain areas of the neurons in loop-like structures is unique to the ZI or if it can be generalized also to neurons of other brain regions, and, lastly, to assess if such coordination plays a facilitatory role in cross-regional communication.

### Conclusions

In this study, our objective was to investigate through cell assembly detection the functional coordination of the ZI with multiple brain areas, which we hypothesize to reflect a cross-regional flux of information. We provided evidence of a highly asymmetrical distribution of probability of detecting pairs in the two directions, namely “from ZI” and “to ZI”. Furthermore, we examined the presence of specific motifs of functional interaction, such as loop-like triplets that suggest a flow of information originating and reentering either in the ZI or in one of the simultaneously recorded regions, with the former being the most common case among the regions considered in the present study. Additionally, incertal neurons that coordinate their activity with all the areas simultaneously recorded, which we interpreted as the units with the highest integrative power, appeared to be the most involved in the formation of loop-like triplets compared to other types of triplets. This could indicate that the loop-like motif may embody a specific, integration-oriented pattern of functional interaction between areas. Future research is needed to determine whether these neurons may contribute to orchestrating the dynamics of the network they are embedded in, similarly to what has been shown for cortical “hub” neurons by recent studies which also highlighted the possibility that some pathological conditions that drive the death of specific neurons may have an impact on the brain network’s dynamics and possibly behavioral implications.^54^^,^^55^

### Limitations of the study

Given the heterogeneity in the afferent and efferent connections^56^ of the incertal neurons recorded in different sectors of the ZI, we should be aware that a limited sampling of the region might result in a partial characterization of its functional interactions. Future studies may examine this issue by systematically sampling multiple sectors of the ZI and contrasting the functional interaction profiles of the cells recorded in these sectors. On the same line, limiting our investigation to ipsilateral inter-area coordination might have biased our reconstruction. Including also contralateral recordings could in future complete the picture of interactions between regions. Moreover, our analysis was limited to triplets because the aim of the present work was to investigate the integrative nature of the ZI, and loop-like assemblies may represent a peculiar and appealing motif of functional interaction. In parallel, the computational cost of extending the analysis to higher-order assemblies was challenging for our computational resources. One of the aims for future studies will certainly be to extend the analyses to more areas and to extend the maximum number of neurons that can participate in the same assembly. Lastly, despite the huge amount of data provided by the dataset by Steinmetz et al.,^35^ some areas are still recorded in only one session, limiting the generalizability of results obtained from data extracted by those single recordings.

A second type of limitation comes when interpreting the nature of the identified functional interactions. We should be aware that the presence of a functional relationship between two regions does not imply causality or a direct drive of one region to the other. The detection of an assembly defined by the delayed activation of two units might in fact flag a variety of scenarios, including a direct (or indirect) excitatory drive of the first unit on the second; an indirect drive of the first unit on the second through double inhibition; a common drive of a third region on both units with different lags; and, finally, the phase locking of both units at different phases of a shared oscillation. While some of these scenarios could be disentangled by further characterizing the activity of the neurons taking part in the assembly, for example, by testing for oscillations and identifying the unit type, a conclusive answer on the nature of the detected interaction might only come from experimental manipulations. The proposed approach can thus provide hypothesis for future experimental investigations. Finally, it is important to highlight that we analyzed the activity recorded in a task requiring the processing of a variety of signals, including visual signals, decision-related information, motor commands, reward-related feedback, and potentially behavioral monitoring signals. The assemblies formed in our study may be thus dependent on the exchange of these types of information. Future studies should address whether different tasks involving, for example, different sensory modalities, may affect the specific pattern of the assemblies formed by ZI.

## STAR★Methods

### Key resources table

**Table:** 

REAGENT or RESOURCE	SOURCE	IDENTIFIER
**Software and algorithms**		

*CADopti*	Russo and Durstewitz^36^ Oettl et al. 2020^13^	https://github.com/DurstewitzLab/CADopti
MATLAB version R2022b and R2023a	MathWorks	https://it.mathworks.com/products/matlab.html
Adobe Illustrator	Adobe	https://www.adobe.com/it/products/illustrator.html

**Other**		

Neurophysiological recordings	Steinmetz et al.^35^	https://figshare.com/articles/steinmetz/9598406

### Resources availability

#### Lead contact

Further information and requests should be directed to the lead contact, Aldo Genovesio (aldo.genovesio@uniroma1.it).

#### Materials availability

This study did not generate new unique reagents.

#### Data and code availability

• This paper analyzes existing, publicly available data. These accession numbers for the datasets are listed in the key resources table.
• This paper does not report original code.
• Any additional information required to reanalyze the data reported in this paper is available from the lead contact upon request.

### Experimental model and study participant details

Our analysis of the ZI was performed by tapping into a public dataset of neuronal recordings made available and described extensively by Steinmetz et al.^35^ Briefly, the recordings were performed in the left hemisphere of 10 mice, possessing heterogeneous genotypes: as reported by Steinmetz et al.^35^ “Ai95; VGlut1-Cre(B6J.Cg-Gt(ROSA)26Sor^tm95.1(CAG−GCaMP6f)Hze^/MwarJ crossed with B6; 129S-Slc17a7 ^tm1.1(cre)Hze^/J), TetO-G6s; Camk2a-tTa (B6; DBA-Tg(tetO-GCaMP6 s)2Niell/J crossed with B6.Cg-Tg(Camk2a-tTA)1Mmay/DboJ), Snap25-G6 s (B6.Cg-Snap25 ^tm3.1Hze^/J), VGlut1-Cre, and wild-type (C57Bl6/J)”.

### Methods details

Recordings collected by Steinmetz et al.^35^ were carried out simultaneously using 2 or 3 neuropixel probes (384 selectable recording sites each) for each recording session. Although our focus was not on the correlation between assembly formation and behavior, and thus task aspects were not considered in the present work, we will give a short description of the tasks used to make clear which task variables can modulate the activity of the neurons forming the assemblies. The mice performed a two-alternative unforced choice task.^57^ Each trial started when the mouse held the wheel for a short interval before the appearance of two visual stimuli (Gabor patch) with different contrasts to the right and left screens. This task required the mice to select the stimulus with higher contrast (Go trials), by turning the wheel that resulted in sliding the chosen stimulus from the peripheral screen to the screen placed in the center. If no stimulus was displayed (NoGo trials), no wheel rotation was required. When the stimuli had the same contrast, the reward was delivered in half of the trials, irrespective of the mouse choice. When only one stimulus was presented, the animal again had to move it to the center of the screen via the wheel. In each recording session, the main task was followed by a passive version of the same task where the same stimuli and trial events of the main task were replayed without requiring any choice by the animal and without the reward delivery. Finally, flashed visual stimuli (white squares) placed in a grid of 10 × 36 positions were randomly presented on a black screen to map receptive fields.^35^

### Quantification and statistical analysis

#### Data analysis

Incertal recordings were collected in four sessions and two mice, together with recordings in a total of 24 other areas, as graphically represented in Figure S1 and reported in Table S1, which represents regions sampled simultaneously in each session with the relative number of neurons recorded. We selected only neurons with more than 100 spikes within the entire recording session, which resulted in 2 neurons of the caudoputamen (CP) being discarded. In total, we analyzed 3067 neurons recorded in all the areas together with the ZI. The labels used to identify the single units reported in Figures 1C, 5B, 5C, 5E and 5F and in section "analysis of loop-like triplets" were arbitrarily defined for this study.

#### Cell assembly detection

For each pair of areas (containing the ZI) or for selected single areas, specifically the ZI and the medial septum (MS), we have applied a cell assembly detection algorithm. Among the many available,^17^ we chose to use *CAD* (Cell Assembly Detection), first developed by Russo and Durstewitz in 2017^36^ and further optimized in its most recent version *CADopti*,^13^ for its flexibility and ability to detect assemblies at any temporal resolution and with any temporal activation pattern. We applied the algorithm to the whole recording, including all the tasks described above. Assembly detection allows, in fact, to investigate neuronal coding without having to anchor the analysis on specific task events or behavioral readout.^58^

*CADopti* is an unsupervised statistical approach that identifies cell assemblies by detecting reoccurring multi-unit activity patterns in spike trains of simultaneously recorded neurons. The algorithm tests if specific multi-unit activity patterns occur more often than what would be expected by chance given the firing statistics of the composing units. The test accounts for non-stationarities, allowing for the investigation of different temporal scales of coding separately. *CADopti* explores a user-selected range of temporal resolutions (bins sizes) and lags in neuronal activity and returns both the optimal temporal resolution and activity pattern of each supra-chance assembly detected. In the present study, we explored the following range of bins and respective maximum lags: BinSizes = [0.01, 0.015, 0.02, 0.025, 0.03, 0.04, 0.05, 0.06, 0.08, 0.1] sec; MaxLags = [19, 12, 9, 7, 6, 4, 3, 2, 2, 1] bins. This choice allowed us to detect assemblies with a characteristic temporal precision smaller than 100 ms and with a maximal lag of 200 ms between the consecutive activation of two neurons.

The term assembly has been used to refer to a variety of forms of correlation, ranging from perfect synchronizations on millisecond scales to sequences of activations spanning tens or hundreds of milliseconds, depending on the brain area of reference and the cognitive process underlying the firing activity.^36^ Since *CADopti* does not require the *a priori* choice of a particular neuronal correlation structure to detect but rather extracts the optimal correlation structure from the data, it stands out as a suitable tool to analyze parallel spike trains generated by the activity of neurons belonging to different brain areas in an unsupervised fashion. In fact, different areas might have different coordination timings due both to different anatomical distances and intrinsic area-specific mechanisms of information processing.

This method consists of two main parts: a pairwise statistical test aiming to quantify the deviation of the joint spike distribution of two neurons from the independence hypothesis, and an agglomerative algorithm that uses this statistical test iteratively to construct assemblies with an arbitrary number of elements.

The goal is therefore to determine if the joint spike count distribution of neurons is significantly distant from that obtained under the null hypothesis of independent processes. Thus, after having binned the parallel spike trains, for each pair of neurons the algorithm counts the number of times the spike of one neuron is followed by a spike of the other after *l* bins. The value for *l* that maximizes the joint spike count is then selected and tested. Statistical testing is performed parametrically, allowing for a quick computation, and corrected for eventual non-stationarities in the time series. Subsequently, the recursive loop begins and the algorithm adds, when possible, a new neuron to the assembly set formed in the previous step, each time considering this set as a new unit to be used in the pairwise test. The iterative algorithm stops when no new neuron can be joined to a previously formed assembly set. This is repeated for all binning values (temporal resolutions) specified by the user, which can be analyzed separately thanks to the non-stationarity corrected statistical test. For more information on the method, see Russo and Durstewitz’s article.^36^

The results reported for assemblies containing only neurons from single areas were obtained by running the algorithm only on neurons from that area, while those for assemblies containing both incertal neurons and neurons from another area were obtained by applying the algorithm to the union of the two sets of neurons recorded in parallel.

#### Definitions

To study the coordination of the ZI with the other simultaneously recorded areas, both for high computational cost and ease of interpretation, we focused on assemblies constituted by two and three neurons, which we will henceforth call pairs and triplets, respectively. Pairs can be sorted into two main categories: the first is formed by cells with a precise 0-lag synchronization (non-directional or synchronous assemblies), and the second is formed by cells with a sequential activation pattern with a delay in the coordinated spiking activity of the assembly units (directional assemblies). When this latter type of assembly includes neurons from two different brain areas, the assembly marks patterns of sequential activation between regions, where the activation of the first assembly neuron, the “leading” neuron, systematically anticipates the activation of another unit (“trailing” neuron) in a different region. Thus, to assess the presence of preferred interaction patterns between regions, we studied the distribution of lags between cross-regional assembly neurons, as done by Oettl et al.^13^ Then, we identified loop-like circuits including a fixed number of three neurons. As for the neural pairs, a loop-like chain of three neurons does not imply an underlying monosynaptic connection between the neurons but highlights a functional coordination. We divided the loop-like triplets detected into two categories: the first one refers to assemblies with a sequential chain of neuronal activation that goes from the ZI to a target area and again to the ZI (ZI→X→ZI); the second one refers to assemblies detected when the first and last neurons of the chain are part of a given area, and the central neuron is an incertal one (X→ZI→X). Because of this particular functional structure, loop-like assemblies might capture feedback or recurrent interaction in the network. Loop-like triplets are always defined by a sequential activation of the units composing the chain. For comparison, we also identified triplets that were not loop-like (henceforth referred to as “non-loop-like triplets”), such as those with same structure as the loop-like triplets but with synchronous activation of at least two units, and those with different neural chains (e.g., X→X→ZI, ZI→ZI→X) that may or may not include neurons with synchronous activation.

Since a different number of neurons were recorded in each area, the number of assemblies identified was evaluated considering the area’s numerosity. Therefore, to compare the capacity of each of the recorded areas to form one of the categories of assemblies described above, we computed the probability obtained by normalizing the number of assemblies returned as significant by the algorithm by the number of possible pairs or triplets of that type that can be formed on that set of elements.

In formulae, let ***A*** and ***B*** be two areas and |***A***| and |***B***| their cardinality, i.e., the number of neurons belonging respectively to the first and second areas, we can define the following quantities: the probability of forming an assembly pair between areas ***A*** and ***B*** as

$$P_{pairs}^{ext}\left(A,B\right)\;≝\;\frac{N_{pairs}^{ext}\left(A,B\right)}{\left|A\right|\cdot \left|B\right|}$$

where $N_{pairs}^{ext}\left(A,B\right)$ is the number of pairs identified by the algorithm and consisting of one neuron of area ***A*** and one neuron of area ***B***; the probability of forming a pair within area ***A*** as

$$P_{pairs}^{\mathrm{int}}\left(A\right)\;≝\;\frac{N_{pairs}^{\mathrm{int}}\left(A\right)}{\left(\begin{array}{c} \left|A\right|\\ 2 \end{array}\right)}$$

where $N_{pairs}^{\mathrm{int}}\left(A\right)$ is the number of pairs internal to ***A*** and therefore consisting of two neurons both belonging to the same area, while $\left(\frac{\left|A\right|}{2}\right)$ is the binomial coefficient that, in this case, identifies all possible pairs of elements belonging to a set of cardinality |***A***|; the probability of forming a loop-like triplet without a specific structure as

$$P_{loop-like}\left(A,B\right)\;≝\;2\cdot \frac{N_{loop-like}\left(A,B\right)}{\left[\left|A\right|\cdot \left|B\right|\cdot \left(\left|A\right|-1\right)\right]+\left[\left|B\right|\cdot \left|A\right|\cdot \left(\left|B\right|-1\right)\right]}$$

where ***N***_***loop***−***like***_(***A***, ***B***) is the number of loop-like triplets with first and last neuron of the chain belonging to one of the two areas, while the intermediate neuron belonging to the other; the probability of forming a loop-like triplet with fixed chain structure ***A*** → ***B*** → ***A*** as

$$P_{loop-like}^{A\to B\to A}\left(A,B\right)\;≝\;2\cdot \frac{N_{loop-like}^{A\to B\to A}\left(A,B\right)}{\left[\left|A\right|\cdot \left|B\right|\cdot \left(\left|A\right|-1\right)\right]}$$

where $N_{loop-like}^{A\to B\to A}\left(A,B\right)$ is the number of loop-like triplets with first and last neuron of the chain belonging to ***A*** and intermediate neuron belonging to ***B***.

Note that if an area was recorded in more than one session, the probabilities described above were computed by dividing the sum of the assemblies identified in the different sessions by the sum of all the possible pairs or triplets of that type that can be formed on each of those sessions.

Analyzing the ability of the ZI to form functional interaction with other brain areas, we were also interested in comparing this ability with that of forming assemblies internally, i.e., consisting only of ZI neurons. To this end, we defined an index indicating how likely it is to form “external assemblies”, i.e., assemblies between the ZI and another area, as opposed to the intrinsic ability to form “internal assemblies”, i.e., assemblies between incertal neurons. Such an “*Int-Ext index”* is the quotient between the probability of forming external assemblies ($P_{pairs}^{ext}$) and the sum of this probability with the probability of forming internal assemblies ($P_{pairs}^{\mathrm{int}}$). As a result, the value of the index varies in the range [0, 1]. A value greater than 0.5 indicates that the probability of forming assemblies between the ZI and another area is greater than the probability of forming assemblies between neurons in the ZI. In contrast, a value less than 0.5 indicates the opposite: it is more likely to form assemblies within the ZI than between the ZI and another area. Finally, a value of exactly 0.5 indicates that the two probabilities are equivalent. In formulae, for each area ***A*** recorded simultaneously with ZI, we have:

$$I_{\mathrm{Int}-Ext}\left(ZI,A\right)\;≝\;\frac{P_{pairs}^{ext}\left(ZI,A\right)}{P_{pairs}^{ext}\left(ZI,A\right)+P_{pairs}^{\mathrm{int}}\left(ZI\right)}$$

Finally, triplets are detected by blocking the assembly agglomeration algorithm at size three. While higher order assemblies, like triplets, by construction stem from significant pair-assemblies, it is not required by the algorithm that all possible pairs of units within an assembly form also a significant assembly pair. In fact, depending on the statistical power, it could occur that two units significantly taking part in a higher-order assembly are slightly below the significance threshold when tested at the pairwise level. This occurred in 46% of the detected loop-like triplets (499/1085). When testing specifically whether the first and second neurons and the second and third neurons of the assembly activation sequence were significant also at the pairwise level, we found that this was the case for 70% of the detected loop-like triplets (741/1085). Figure S8 shows the same analysis displayed in Figure 6 when restricted to this set of triplets. The results displayed in the two figures are highly comparable. This suggests that the triplets for which either the first-second unit pair or the second-third unit pair was not significant, are not different in nature from the rest of the loop-like triplets and, as expected, the absence of pairwise significance is to be attributed to a lack of statistical power. To avoid reducing the sample size, all following analyses are performed including all loop-like triplets.

#### Graph-based representation

We employed a graph-based representation to illustrate the coordinative relationships of the ZI, both internally and with other brain areas. A first graph was generated to describe the coordination of the ZI with the other areas recorded simultaneously (Figure 1B). To compute this oriented graph, we used MATLAB’s function *"digraph"*. A central node representing the ZI was surrounded by a group of nodes corresponding the other areas in a circular arrangement. If a directional assembly was formed between ZI and another area, the two nodes were connected by an edge. The edges were directional, and their thickness was proportional to the probability of forming an assembly with that directionality.

We also generated a graph from an example session (Figure 2) to show the internal coordination of the ZI neurons with each other and with the other neurons of the external areas. Each blue node of the graph represents a ZI neuron, while each orange node identifies an external area recorded simultaneously with the ZI. We placed an edge between two ZI neurons if they belonged to the same assembly, or between one ZI neuron and another area if at least one assembly was formed with neurons of that specific area. In this figure, to avoid adding unnecessary complexity, we neglected the directionality of the edges and displayed ZI interregional coordination as a non-oriented graph. We used MATLAB’s function “*graph*” to generate this latter graph, and for the spatial disposition of the nodes we imposed the use of the Force-directed Placement algorithm,^59^ which simulates an attractive force between adjacent nodes and a repulsive force between distant ones.

Finally, another non-oriented graph but with the same circular arrangement as the one in Figure 1B was generated to represent the probability of forming synchronous assemblies between ZI and each of the other areas (Figure S2).

#### Circular shuffling

To validate the results obtained and compare the number of assemblies returned by the algorithm on the recorded data with the number that would be obtained on shuffled data, we constructed surrogate datasets by bootstrapping the spike trains of the recorded neurons. Similar to the approach used in previous studies,^60^^,^^61^ we shifted the complete activity of each neuron circularly over time. The amount of the shift was determined randomly and independently for each neuron. Note that this procedure preserves the overall firing rate and the internal temporal structure of each neuron, while destroying cross-correlation between neurons and thus randomizing co-activations. These surrogate datasets were then analyzed with the *CADopti* algorithm for comparison with the recorded data. The circular shuffling procedure was repeated 10 times. The results obtained were averaged over the runs and normalized with respect to the number of possible pairs of elements on the set of neurons considered.

#### Statistical tests

To assess the statistical significance of the asymmetries in the probability of detecting assemblies of two neurons with different directionality, we performed a binomial test on the number of cell assemblies in one specific direction over the total number of directional assemblies. The two possible directions were considered equiprobable; therefore, a probability of 0.5 was assigned under the null hypothesis.

Similarly, at the level of the loop-like triplets, we assessed the statistical significance of the directional asymmetry using a binomial test. However, in this case the two possible chains of neuronal activation were not considered to be equiprobable as the number of recorded neurons in the two regions affects the probability of the type of structure. For example, if only two neurons are recorded in the ZI and one neuron in another area, the only loop-like assembly that can be detected is of the form ZI→X→ZI. This implies a dependence of the number of possible loop-like triplets formed in the two chains of activation on the number of neurons recorded in the ZI and in the other areas. Thus, under the null hypothesis, we have assigned to each type of loop-like triplet a probability equal to the number of possible triplets of that type obtainable with that set of neurons, over the number of possible triplets in which one neuron belongs to one area and the other two to the other area. In formulae, for two generic areas ***A*** and ***B***:

$$P_{expected}\left(A\to B\to A\right)=\frac{\left|A\right|\cdot \left|B\right|\cdot \left(\left|A\right|-1\right)}{\left[\left|A\right|\cdot \left|B\right|\cdot \left(\left|A\right|-1\right)\right]+\left[\left|B\right|\cdot \left|A\right|\cdot \left(\left|B\right|-1\right)\right]}=\frac{\left|A\right|-1}{\left|A\right|+\left|B\right|-2}$$

Because of an excessively low number of assemblies detected, some areas were not tested for a preference in forming specific assembly type. Tests were performed only on areas with at least 10 directional assemblies, in the case of pairs, and at least 10 loop-like assemblies in the case of the triplets. Using this threshold, 4 areas were excluded from the statistics on directional asymmetry at the pair level, while 11 areas were excluded from the statistical testing of the asymmetry of the two possible structures of loop-like triplets.

## References

[1] Mitrofanis J. (2005). Some certainty for the “zone of uncertainty”? Exploring the function of the zona incerta. Neuroscience.

[2] Yang Y., Jiang T., Jia X., Yuan J., Li X., Gong H. (2022). Whole-Brain Connectome of GABAergic Neurons in the Mouse Zona Incerta. Neurosci. Bull..

[3] Wang X., Chou X.L., Zhang L.I., Tao H.W. (2020). Zona Incerta: An Integrative Node for Global Behavioral Modulation. Trends Neurosci..

[4] Plaha P., Ben-Shlomo Y., Patel N.K., Gill S.S. (2006). Stimulation of the caudal zona incerta is superior to stimulation of the subthalamic nucleus in improving contralateral parkinsonism. Brain.

[5] Díaz-Parra A., Osborn Z., Canals S., Moratal D., Sporns O. (2017). Structural and functional, empirical and modeled connectivity in the cerebral cortex of the rat. Neuroimage.

[6] Hirabayashi T., Takeuchi D., Tamura K., Miyashita Y. (2013). Functional Microcircuit Recruited during Retrieval of Object Association Memory in Monkey Perirhinal Cortex. Neuron.

[7] Sakurai Y., Nakazono T., Ishino S., Terada S., Yamaguchi K., Takahashi S. (2013). Diverse synchrony of firing reflects diverse cell-assembly coding in the prefrontal cortex. J. Physiol. Paris.

[8] Deolindo C.S., Kunicki A.C.B., Da Silva M.I., Lima Brasil F., Moioli R.C. (2017). Neuronal Assemblies Evidence Distributed Interactions within a Tactile Discrimination Task in Rats. Front. Neural Circ..

[9] Siegle J.H., Jia X., Durand S., Gale S., Bennett C., Graddis N., Heller G., Ramirez T.K., Choi H., Luviano J.A. (2021). Survey of spiking in the mouse visual system reveals functional hierarchy. Nature.

[10] Kreuz T., Haas J.S., Morelli A., Abarbanel H.D.I., Politi A. (2007). Measuring spike train synchrony. J. Neurosci. Methods.

[11] Bastos A.M., Schoffelen J.-M. (2015). A Tutorial Review of Functional Connectivity Analysis Methods and Their Interpretational Pitfalls. Front. Syst. Neurosci..

[12] Battaglia D., Brovelli A., Poeppel D., Mangun G.R., Gazzaniga M.S. (2020). The Cognitive Neurosciences.

[13] Oettl L.-L., Scheller M., Filosa C., Wieland S., Haag F., Loeb C., Durstewitz D., Shusterman R., Russo E., Kelsch W. (2020). Phasic dopamine reinforces distinct striatal stimulus encoding in the olfactory tubercle driving dopaminergic reward prediction. Nat. Commun..

[14] Domanski A.P.F., Kucewicz M.T., Russo E., Tricklebank M.D., Robinson E.S.J., Durstewitz D., Jones M.W. (2023). Distinct hippocampal-prefrontal neural assemblies coordinate memory encoding, maintenance, and recall. Curr. Biol..

[15] Hebb D.O. (2005).

[16] Lebedev M.A., Nicolelis M.A.L. (2017). Brain-Machine Interfaces: From Basic Science to Neuroprostheses and Neurorehabilitation. Physiol. Rev..

[17] Quaglio P., Rostami V., Torre E., Grün S. (2018). Methods for identification of spike patterns in massively parallel spike trains. Biol. Cybern..

[18] Riehle A., Grün S., Diesmann M., Aertsen A. (1997). Spike Synchronization and Rate Modulation Differentially Involved in Motor Cortical Function. Science.

[19] Funahashi S., Inoue M. (2000). Neuronal Interactions Related to Working Memory Processes in the Primate Prefrontal Cortex Revealed by Cross-correlation Analysis. Cerebr. Cortex.

[20] Constantinidis C., Franowicz M.N., Goldman-Rakic P.S. (2001). Coding Specificity in Cortical Microcircuits: A Multiple-Electrode Analysis of Primate Prefrontal Cortex. J. Neurosci..

[21] Tsujimoto S., Genovesio A., Wise S.P. (2008). Transient Neuronal Correlations Underlying Goal Selection and Maintenance in Prefrontal Cortex. Cerebr. Cortex.

[22] Nougaret S., Genovesio A. (2018). Learning the meaning of new stimuli increases the cross-correlated activity of prefrontal neurons. Sci. Rep..

[23] Mione V., Tsujimoto S., Genovesio A. (2019). Neural Correlations Underlying Self-Generated Decision in the Frontal Pole Cortex during a Cued Strategy Task. Neuroscience.

[24] Jun J.J., Steinmetz N.A., Siegle J.H., Denman D.J., Bauza M., Barbarits B., Lee A.K., Anastassiou C.A., Andrei A., Aydın Ç. (2017). Fully integrated silicon probes for high-density recording of neural activity. Nature.

[25] Steinmetz N.A., Aydin C., Lebedeva A., Okun M., Pachitariu M., Bauza M., Beau M., Bhagat J., Böhm C., Broux M. (2021). Neuropixels 2.0: A miniaturized high-density probe for stable, long-term brain recordings. Science.

[26] Milo R., Shen-Orr S., Itzkovitz S., Kashtan N., Chklovskii D., Alon U. (2002). Network Motifs: Simple Building Blocks of Complex Networks. Science.

[27] Choi J., Lee D. (2018). Topological motifs populate complex networks through grouped attachment. Sci. Rep..

[28] Sporns O., Kötter R. (2004). Motifs in Brain Networks. PLoS Biol..

[29] Dechery J.B., MacLean J.N. (2018). Functional triplet motifs underlie accurate predictions of single-trial responses in populations of tuned and untuned V1 neurons. PLoS Comput. Biol..

[30] Panzeri S., Moroni M., Safaai H., Harvey C.D. (2022). The structures and functions of correlations in neural population codes. Nat. Rev. Neurosci..

[31] Hulse B.K., Haberkern H., Franconville R., Turner-Evans D., Takemura S.Y., Wolff T., Noorman M., Dreher M., Dan C., Parekh R. (2021). A connectome of the Drosophila central complex reveals network motifs suitable for flexible navigation and context-dependent action selection. Elife.

[32] Alon U. (2007). Network motifs: theory and experimental approaches. Nat. Rev. Genet..

[33] Wei Y., Liao X., Yan C., He Y., Xia M. (2017). Identifying topological motif patterns of human brain functional networks. Hum. Brain Mapp..

[34] Duclos C., Nadin D., Mahdid Y., Tarnal V., Picton P., Vanini G., Golmirzaie G., Janke E., Avidan M.S., Kelz M.B. (2021). Brain network motifs are markers of loss and recovery of consciousness. Sci. Rep..

[35] Steinmetz N.A., Zatka-Haas P., Carandini M., Harris K.D. (2019). Distributed coding of choice, action and engagement across the mouse brain. Nature.

[36] Russo E., Durstewitz D. (2017). Cell assemblies at multiple time scales with arbitrary lag constellations. Elife.

[37] May P.J., Basso M.A. (2018). Connections between the zona incerta and superior colliculus in the monkey and squirrel. Brain Struct. Funct..

[38] Watson G.D.R., Smith J.B., Alloway K.D. (2015). The Zona Incerta Regulates Communication between the Superior Colliculus and the Posteromedial Thalamus: Implications for Thalamic Interactions with the Dorsolateral Striatum. J. Neurosci..

[39] Ficalora A.S., Mize R.R. (1989). The neurons of the substantia nigra and zona incerta which project to the cat superior colliculus are GABA immunoreactive: A double-label study using GABA immunocytochemistry and lectin retrograde transport. Neuroscience.

[40] Bolton A.D., Murata Y., Kirchner R., Kim S.-Y., Young A., Dang T., Yanagawa Y., Constantine-Paton M. (2015). A Diencephalic Dopamine Source Provides Input to the Superior Colliculus, where D1 and D2 Receptors Segregate to Distinct Functional Zones. Cell Rep..

[41] Chou X.L., Wang X., Zhang Z.G., Shen L., Zingg B., Huang J., Zhong W., Mesik L., Zhang L.I., Tao H.W. (2018). Inhibitory gain modulation of defense behaviors by zona incerta. Nat. Commun..

[42] Mitrofanis J. (2002). Distinctive patterns of connectivity between the zona incerta and the red nucleus of rats. Anat. Embryol..

[43] Heise C.E., Mitrofanis J. (2004). Evidence for a glutamatergic projection from the zona incerta to the basal ganglia of rats. J. Comp. Neurol..

[44] Li L.-X., Li Y.-L., Wu J.-T., Song J.-Z., Li X.-M. (2022). Glutamatergic Neurons in the Caudal Zona Incerta Regulate Parkinsonian Motor Symptoms in Mice. Neurosci. Bull..

[45] Power B.D., Kolmac C.I., Mitrofanis J. (1999). Evidence for a large projection from the zona incerta to the dorsal thalamus. J. Comp. Neurol..

[46] Paxinos G. (2004).

[47] Barthó P., Freund T.F., Acsády L. (2002). Selective GABAergic innervation of thalamic nuclei from zona incerta. Eur. J. Neurosci..

[48] Trageser J.C., Burke K.A., Masri R., Li Y., Sellers L., Keller A. (2006). State-Dependent Gating of Sensory Inputs by Zona Incerta. J. Neurophysiol..

[49] Zhou M., Liu Z., Melin M.D., Ng Y.H., Xu W., Südhof T.C. (2018). A central amygdala to zona incerta projection is required for acquisition and remote recall of conditioned fear memory. Nat. Neurosci..

[50] Zhao Z.D., Chen Z., Xiang X., Hu M., Xie H., Jia X., Cai F., Cui Y., Chen Z., Qian L. (2019). Zona incerta GABAergic neurons integrate prey-related sensory signals and induce an appetitive drive to promote hunting. Nat. Neurosci..

[51] Riley J.N., Moore R.Y. (1981). Diencephalic and brainstem afferents to the hippocampal formation of the rat. Brain Res. Bull..

[52] Bittencourt J.C., Elias C.F. (1998). Melanin-concentrating hormone and neuropeptide EI projections from the lateral hypothalamic area and zona incerta to the medial septal nucleus and spinal cord: a study using multiple neuronal tracers. Brain Res..

[53] Edelman G.M., Gally J.A. (2013). Reentry: a key mechanism for integration of brain function. Front. Integr. Neurosci..

[54] Gal E., Amsalem O., Schindel A., London M., Schürmann F., Markram H., Segev I. (2021). The Role of Hub Neurons in Modulating Cortical Dynamics. Front. Neural Circ..

[55] Gal E., London M., Globerson A., Ramaswamy S., Reimann M.W., Muller E., Markram H., Segev I. (2017). Rich cell-type-specific network topology in neocortical microcircuitry. Nat. Neurosci..

[56] Arena G., Londei F., Ceccarelli F., Ferrucci L., Borra E., Genovesio A. (2023). Disentangling the identity of the zona incerta: a review of the known connections and latest implications. Ageing Res. Rev..

[57] Burgess C.P., Lak A., Steinmetz N.A., Zatka-Haas P., Bai Reddy C., Jacobs E.A.K., Linden J.F., Paton J.J., Ranson A., Schröder S. (2017). High-Yield Methods for Accurate Two-Alternative Visual Psychophysics in Head-Fixed Mice. Cell Rep..

[58] Hunt L.T., Daw N.D., Kaanders P., MacIver M.A., Mugan U., Procyk E., Redish A.D., Russo E., Scholl J., Stachenfeld K. (2021). Formalizing planning and information search in naturalistic decision-making. Nat. Neurosci..

[59] Fruchterman T.M.J., Reingold E.M. (1991). Graph drawing by force-directed placement. Software Pract. Ex..

[60] Benozzo D., La Camera G., Genovesio A. (2021). Slower prefrontal metastable dynamics during deliberation predicts error trials in a distance discrimination task. Cell Rep..

[61] Maboudi K., Ackermann E., De Jong L.W., Pfeiffer B.E., Foster D., Diba K., Kemere C. (2018). Uncovering temporal structure in hippocampal output patterns. Elife.

