# Supplementary material for: Connecting the dots in the zona incerta: A study of neural assemblies and motifs of inter-area coordination in mice
Source: iScience. 2023 Dec 16;27(1):108761. doi: 10.1016/j.isci.2023.108761 (PMC10808920; doi:10.1016/j.isci.2023.108761)
Supplement: Document S1. Figures S1–S8 and Tables S1–S5 [file mmc1.pdf]

iScience, Volume *27*

Supplemental information

Connecting the dots in the zona incerta: A study of neural assemblies and motifs of inter-area coordination in mice

Fabrizio Londei, Giulia Arena, Lorenzo Ferrucci, Eleonora Russo, Francesco Ceccarelli, and Aldo Genovesio

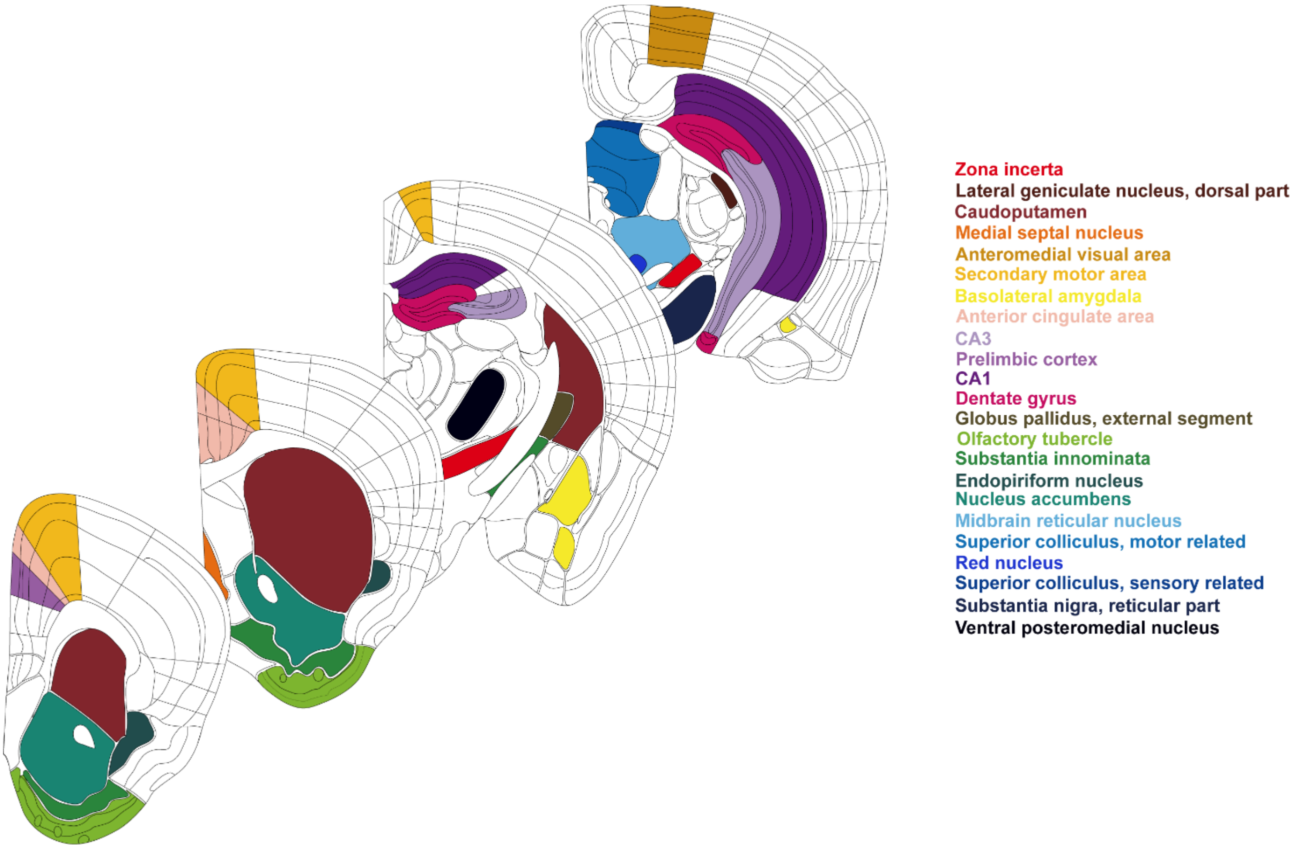

Figure S1. Anatomical representation of considered areas, related to STAR Methods.

Areas (with color-coded labels) included in the analysis performed in the present work.

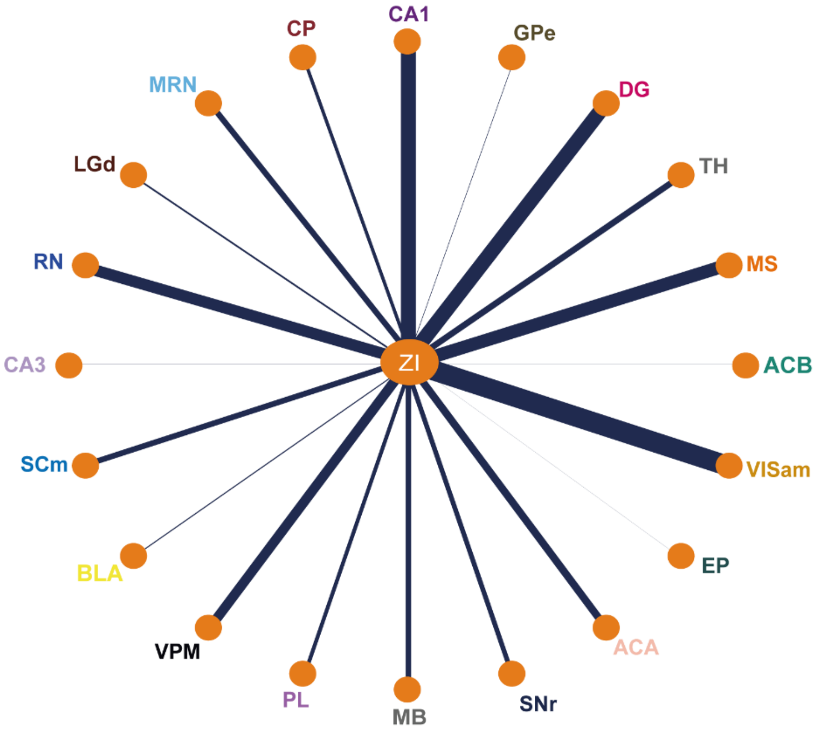

Figure S2. Probability of forming non-directional pairs, related to Figure 1.

Graph representing the probability of the ZI to form non-directional pairs with the external regions. The thickness of the edges is proportional to such probability.

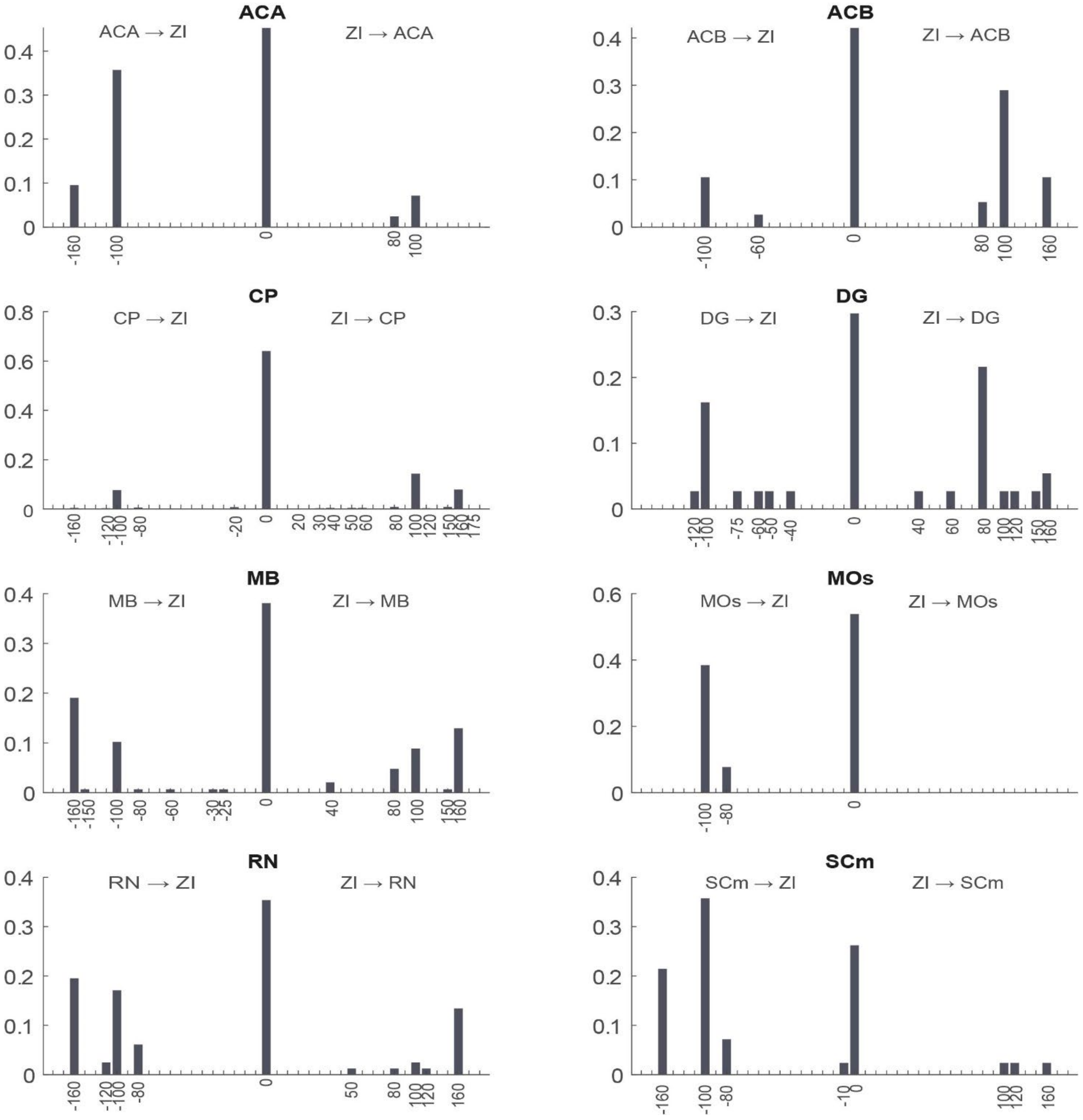

Figure S3. Distribution of latencies in couples ZI/ ACA, ACB, CP, DG, MB, MOs, RN, SCm, related to STAR Methods.

Distribution of the latencies in activation of incertal neurons forming assemblies with the following areas: ACA, ACB, CP, DG, MB, MOs, RN, SCm. Positive values correspond to assemblies in which the incertal unit activates first, negative values vice versa. To be noted, in these histograms we pooled latencies information from all assemblies, also when detected at different temporal resolutions.

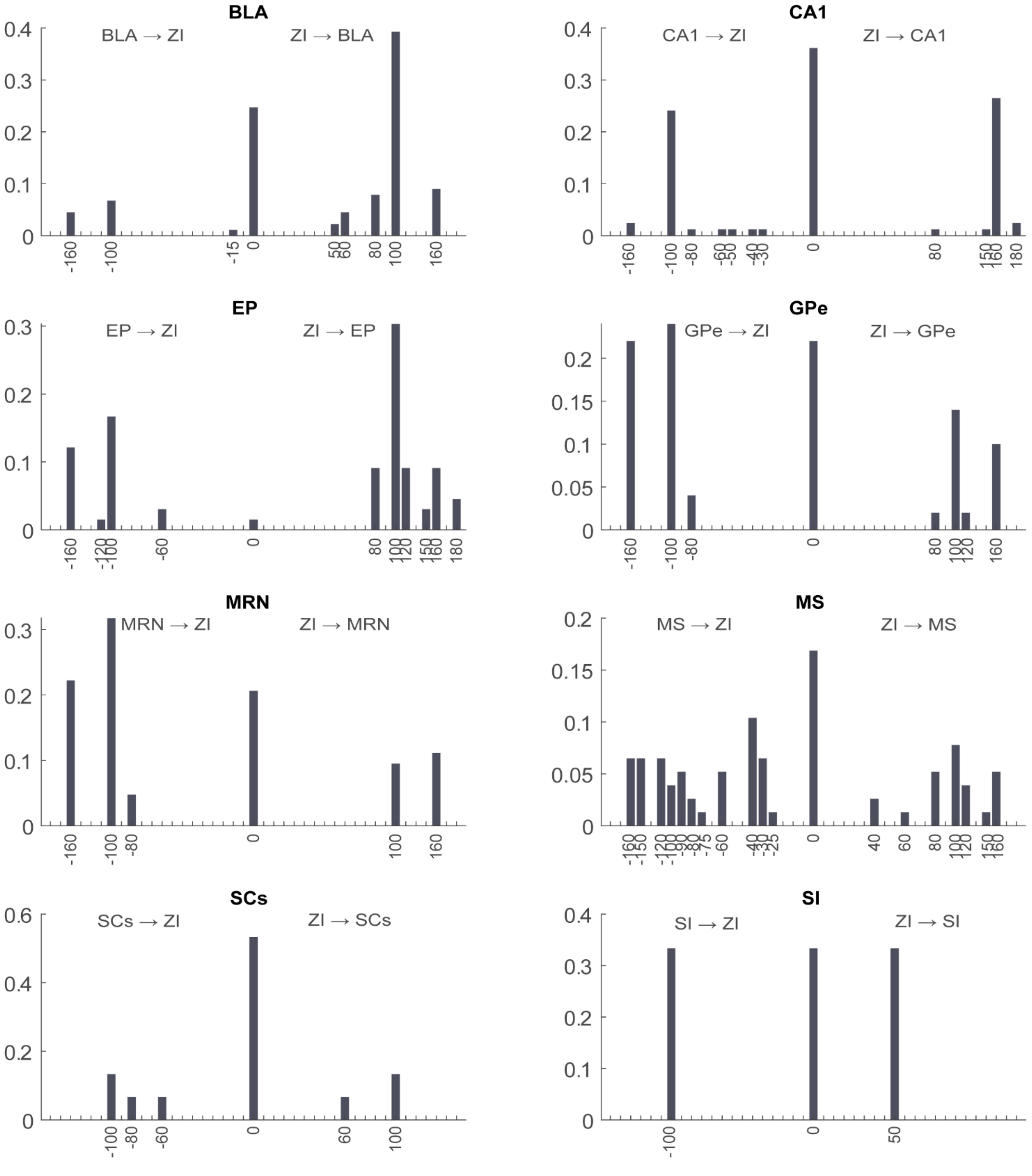

Figure S4. Distribution of latencies in couples ZI/ BLA, CA1, EP, GPe, MRN, MS, SCs, SI, related to STAR Methods.

Distribution of the latencies in activation of incertal neurons forming assemblies with the following areas: BLA, CA1, EP, GPe, MRN, MS, SCs, SI. Positive values correspond to assemblies in which the incertal unit activates first, negative values vice versa. To be noted, in these histograms we pooled latencies information from all assemblies, also when detected at different temporal resolutions.

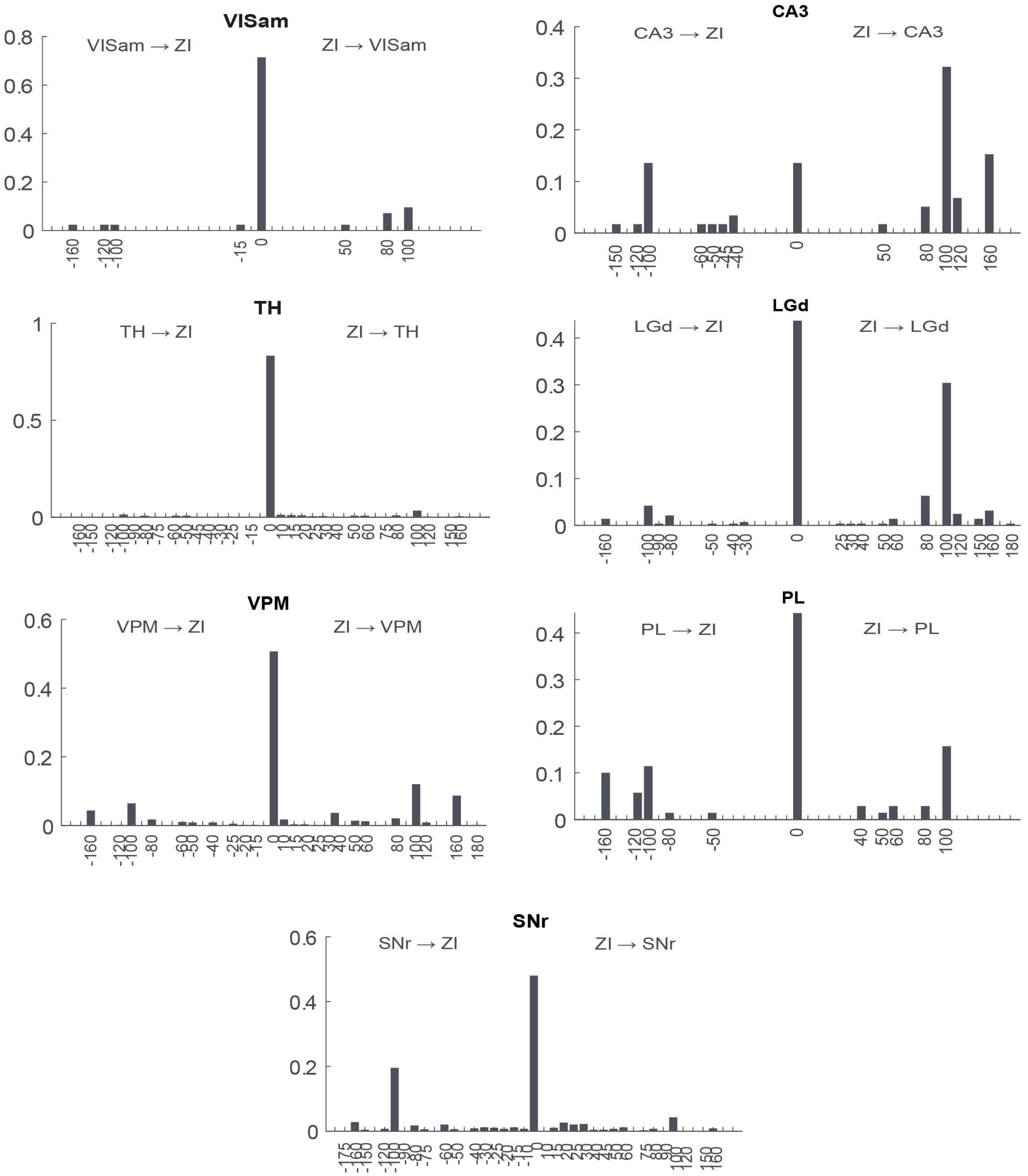

Figure S5. Distribution of latencies in couples ZI/ VISam, CA3, TH, LGd, VPM, PL, SNr, related to STAR Methods.

Distribution of the latencies in activation of incertal neurons forming assemblies with the following areas: VISam, CA3, TH, LGd, VPM, PL, SNr. Positive values correspond to assemblies in which the incertal unit activates first, negative values vice versa. To be noted, in these histograms we pooled latencies information from all assemblies, also when detected at different temporal resolutions.

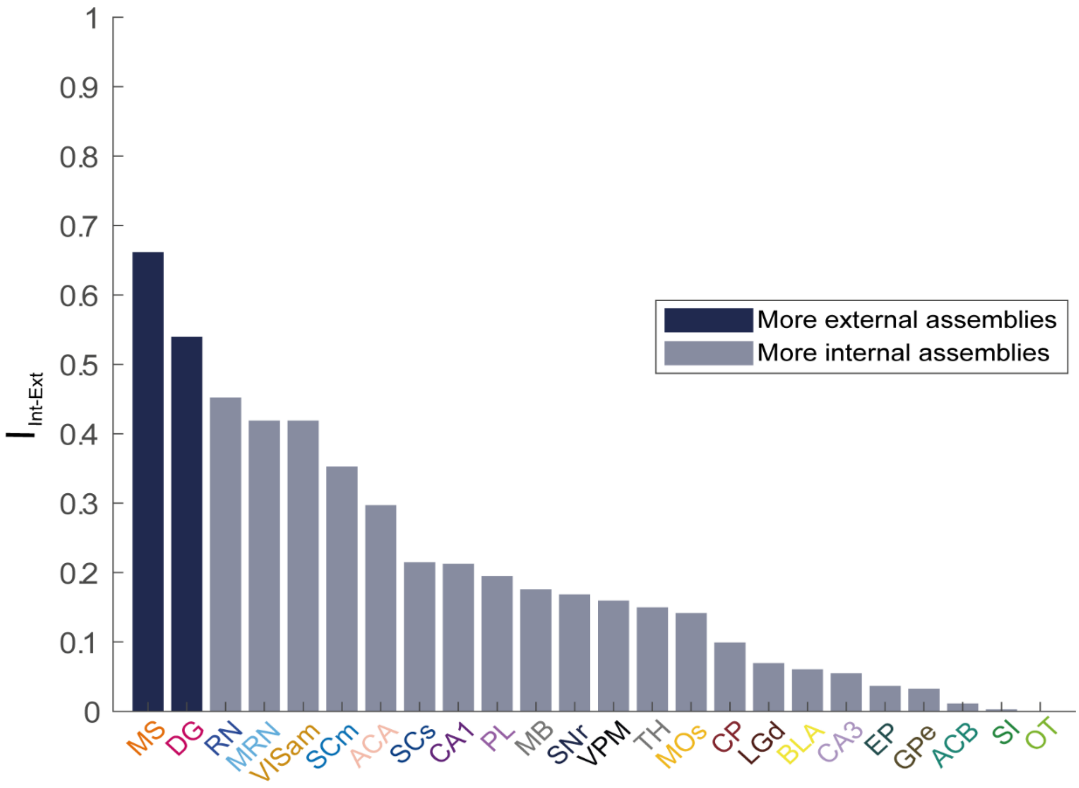

Figure S6. Int-Ext index ranking of areas, related to STAR Methods.

Int-Ext index computed including the internal assemblies of the ZI and the external assemblies the ZI forms with region X. On the x-axis is reported the identity of X.

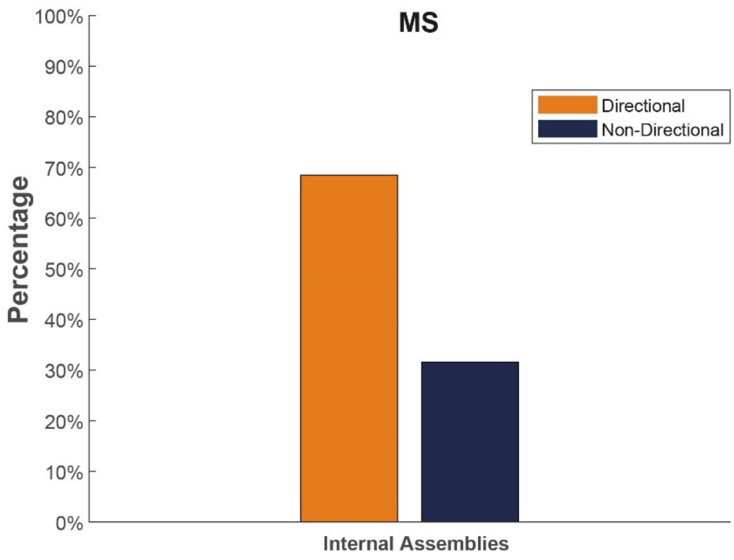

Figure S7. Cell assembly detection on the MS, related to Figure 3.

Histogram showing the percentage of detected directional or non-directional assemblies internal to MS.

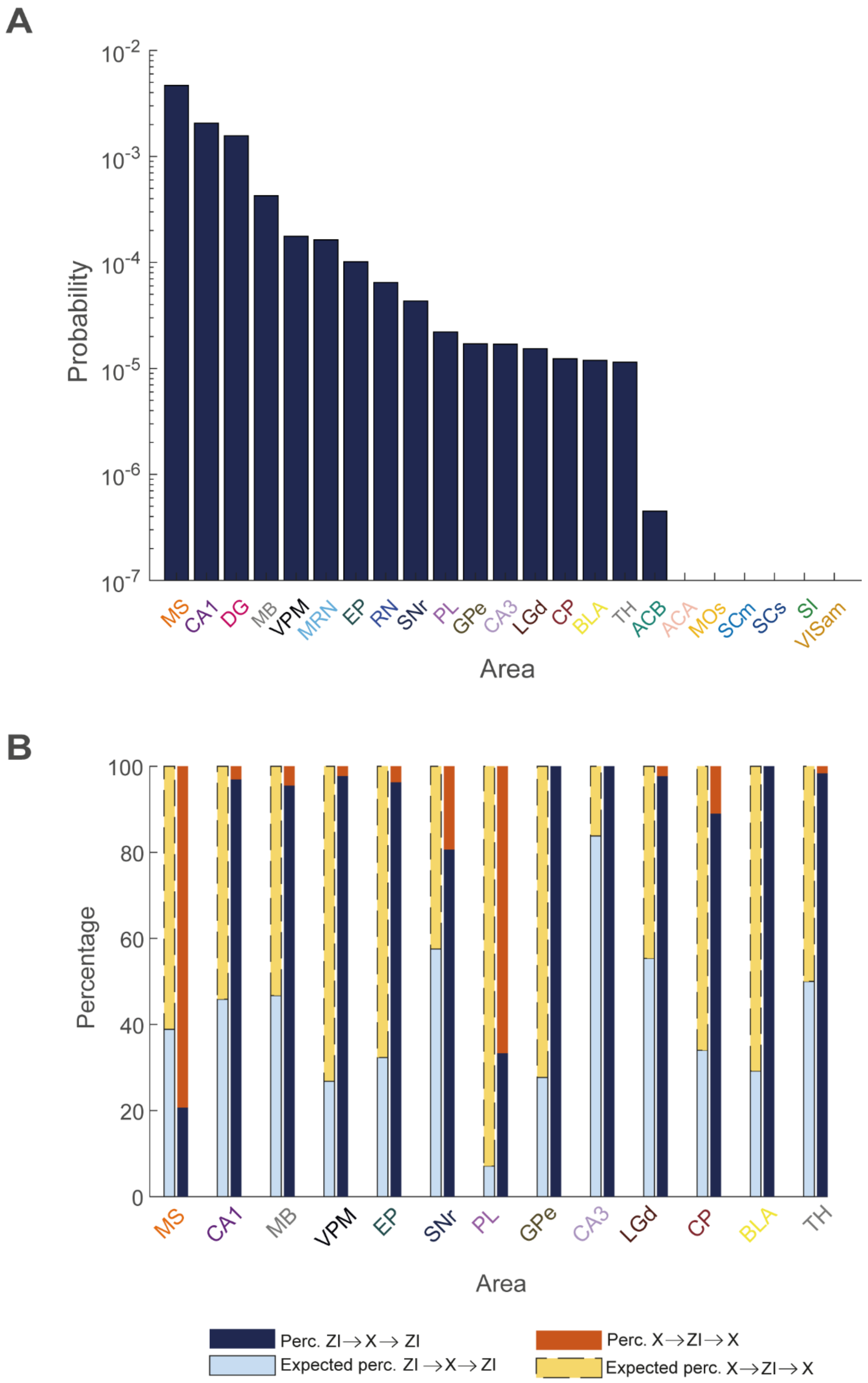

Figure S8. Loop-like analysis on a restricted set of triplets, related to Figure 6.

Same as Fig. 6 but restricting the analysis on loop-like triplets in which assembly significance is present both at the triplet level as well as at the pairwise level between the first-second neurons and the second-third neurons within the activation chain.

Session 1		Session 2		Session 3		Session 4	
Recorded Areas	*Number of neurons*	*Recorded areas*	*Number of neurons*	*Recorded areas*	*Number of neurons*	*Recorded areas*	*Number of neurons*
ZI	15	***ZI***	55	***ZI***	175	***ZI***	44
ACA	58	***BLA***	132	***ACB***	155	***CA1***	4
CA1	34	***CA3***	3	***CA3***	35	***CA3***	7
DG	17	***GPe***	142	***CP***	157	***CP***	382
LGd	150	***LGd***	121	***LGd***	89	***EP***	91
MB	63	***MB***	45	***OT***	26	***LGd***	34
MOs	46	***VPM***	162	***SI***	48	***SNr***	15
MRN	51			***SNr***	130	***VPM***	31
MS	23			***TH***	175		
PL	184						
RN	58						
SCm	45						
SCs	32						
VISam	34						

Table S1. Number of neurons recorded for each considered area in each session, related to STAR Methods.

Areas simultaneously recorded in each session along with the number of units recorded in each area, after the removal of units with less than 100 spikes throughout the entire recording session. Sessions 1, 2, and 3 were recorded on mouse 1, session 4 was recorded on mouse 2.

Couple ZI/	Pairs	
	p-value	Preferred direction
ACA	0.0025995	To ZI
ACB	0.016901	From ZI
BLA	2.1458e-08	From ZI
CA1	1	To ZI
CA3	0.0046013	From ZI
CP	5.336e-13	From ZI
DG	0.5572	From ZI
EP	0.012503	From ZI
GPe	0.10813	To ZI
LGd	1.4918e-18	From ZI
MB	0.67522	To ZI
MRN	0.00093622	To ZI
MS	0.0081469	To ZI
PL	0.74926	To ZI
RN	0.0054863	To ZI
SCm	4.6492e-06	To ZI
SNr	6.9773e-10	To ZI
TH	0.00017238	From ZI
VISam	0.3877	From ZI
VPM	1.705e-08	From ZI

Table S2. Preferred direction of functional interaction with the associated p-value, related to STAR Methods.

Statistical significance, computed with a binomial test, of the asymmetry in the probability to detect pairs with a specific directionality between ZI and each area mentioned in the left column. Right column indicates the preferred directionality that corresponds to the directionality that counts the highest number of assemblies in a specific couple. Areas not included do not meet the selection criterion based on the number of assemblies formed (see *STAR Methods* section).

Couple ZI/	Neurons	Pairs			
		From ZI	To ZI	Non-directional	Total
ACA	58	4 (0.0045977)	19 (0.021839)	19 (0.021839)	42
ACB	155	17 (6.2673e-04)	5 (1.8433e-04)	16 (5.8986e-04)	38
BLA	132	56 (0.0077135)	11 (0.0015152)	22 (0.0030303)	89
CA1	38	26 (0.037901)	27 (0.039359)	30 (0.043732)	83
CA3	45	36 (0.0054562)	15 (0.0022734)	8 (0.0012125)	59
CP	539	176 (0.0039744)	65 (0.0014678)	429 (0.0096877)	670
DG	17	15 (0.058824)	11 (0.043137)	11 (0.043137)	37
EP	91	43 (0.010739)	22 (0.0054945)	1 (2.4975e-04)	66
GPe	142	14 (0.0017926)	25 (0.003201)	11 (0.0014085)	50
LGd	394	134 (0.0051586)	27 (0.0010394)	125 (0.0048121)	286
MB	108	43 (0.012573)	48 (0.014035)	56 (0.016374)	147
MOs	46	0	6 (0.0086957)	7 (0.010145)	13
MRN	51	13 (0.016993)	37 (0.048366)	13 (0.016993)	63
MS	23	21 (0.06087)	43 (0.12464)	13 (0.037681)	77
OT	26	0	0	0	0
PL	184	18 (0.0065217)	21 (0.0076087)	31 (0.011232)	70
RN	58	16 (0.018391)	37 (0.042529)	29 (0.033333)	82
SCm	45	3 (0.0044444)	28 (0.041481)	11 (0.016296)	42
SCs	32	3 (0.00625)	4 (0.0083333)	8 (0.016667)	15
SI	48	1 (1.1905e-04)	1 (1.1905e-04)	1 (1.1905e-04)	3
SNr	145	119 (0.0050833)	235 (0.010038)	327 (0.013968)	681
TH	175	75 (0.002449)	35 (0.0011429)	547 (0.017861)	657
VISam	34	8 (0.015686)	4 (0.0078431)	30 (0.058824)	42
VPM	193	189 (0.018396)	94 (0.0091493)	291 (0.028324)	574

Table S3. Number of detected assemblies between ZI and each one of the other areas, related to STAR Methods.

The “Neurons” column indicates the number of cells recorded for each area that forms pair assemblies with ZI. The first three columns of “Pairs” refer to the number of assemblies of each area sorted on the basis of their directionality in “from ZI”, “to ZI” and “non-directional”. In parenthesis the correspondent probability of occurrence given the recorded neurons in each region. The rightmost column report to the total number of assemblies formed by each area with ZI.

Couple ZI/	Loop-like triplets	
	**p-value**	**Preferred structure**
BLA	3.0563e-10	ZI→BLA→ZI
CA1	3.4153e-09	ZI→CA1→ZI
CA3	0.0996	ZI→CA3→ZI
CP	9.9077e-23	ZI→CP→ZI
EP	4.1411e-14	ZI→EP→ZI
GPe	2.3296e-21	ZI→GPe→ZI
LGd	1.1409e-15	ZI→LGd→ZI
MB	9.5602e-25	ZI→MB→ZI
MS	1.3732e-05	MS→ZI→MS
PL	0.1555	PL→ZI→PL
SNr	7.0193e-06	ZI→SNr→ZI
TH	3.9133e-14	ZI→TH→ZI
VPM	4.5654e-36	ZI→VPM→ZI

Table S4. Preferred loop-like structure with the associated p-value, related to STAR Methods.

Statistical significance of a binomial test, computed to assess the asymmetry in probability of forming ZI→X→ZI or X→ZI→X loop-like triplets for each area reported in the left column with ZI. The preferred type of loop-like structure is reported in the rightmost column. Areas not included do not meet the selection criterion explained in the *STAR Methods* section.

Couple ZI/	Loop-like triplets	
	ZI->X->ZI	X->ZI->X
ACA	0	0
ACB	2 (8.4750e-07)	0
BLA	20 (1.0203e-04)	1 (2.1029e-06)
CA1	32 (0.0044)	2 (2.3044e-04)
CA3	18 (3.3091e-05)	0
CP	99 (3.5978e-05)	23 (4.3031e-06)
DG	6 (0.0034)	0
EP	30 (3.4849e-04)	1 (5.5500e-06)
GPe	37 (1.7546e-04)	0
LGd	73 (4.6126e-05)	1 (7.8314e-07)
MB	95 (0.0013)	6 (7.1646e-05)
MOs	0	0
MRN	2 (3.7348e-04)	6 (3.1373e-04)
MS	6 (0.0025)	47 (0.0124)
PL	2 (1.0352e-04)	8 (3.1678e-05)
RN	0	5 (2.0165e-04)
SCm	0	0
SCs	0	0
SI	0	0
SNr	170 (8.5280e-05)	67 (4.5516e-05)
TH	61 (2.2895e-05)	4 (1.5013e-06)
VISam	0	0
VPM	245 (9.0776e-04)	16 (2.1689e-05)

Table S5. Number of detected loop-like triplets between ZI and each one of the other areas, related to STAR Methods.

Number and, in parenthesis, relative probabilities of the loop-like triplets detected and sorted into the two possible structures. OT is not included since no pairs have been detected and no analysis on triplets was performed.
